# Optimization of Ultra-High-Performance Liquid Chromatography-Electrospray Ionization-Mass Spectrometry Detection of Glutamine-FMOC Ad-Hoc Derivative by Central Composite Design

**DOI:** 10.1038/s41598-020-64099-w

**Published:** 2020-04-28

**Authors:** Khaggeswar Bheemanapally, Mostafa M. H. Ibrahim, Karen P. Briski

**Affiliations:** School of Basic Pharmaceutical and Toxicological Sciences, College of Pharmacy, University of Louisiana at Monroe, Monroe, LA 71201 United States

**Keywords:** Mass spectrometry, Neurology

## Abstract

Glutamine (Gln) is converted to excitatory (glutamate, aspartate) and inhibitory (γ-amino butyric acid) amino acid neurotransmitters in brain, and is a source of energy during glucose deprivation. Current research utilized an Analytical Quality by Design approach to optimize levels and combinations of critical gas pressure (sheath, auxiliary, sweep) and temperature (ion transfer tube, vaporizer) parameters for high-sensitivity mass spectrometric quantification of brain tissue glutamine. A Design of Experiments (DOE) matrix for evaluation of relationships between these multiple independent variables and a singular response variable, e.g. glutamine chromatogram area, was developed by statistical response surface methodology using central composite design. A second-order polynomial equation was generated to identify and predict singular versus combinatory effects of synergistic and antagonistic factors on chromatograph area. Predicted versus found outcomes overlapped, with enhanced area associated with the latter. DOE methodology was subsequently used to evaluate liquid chromatographic variable effects, e.g. flow rate, column temperature, and mobile phase composition on the response variable. Results demonstrate that combinatory AQbD-guided mass spectrometric/liquid chromatographic optimization significantly enhanced analytical sensitivity for Gln, thus enabling down-sized brain tissue sample volume procurement for quantification of this critical amino acid.

## Introduction

There is increasing appreciation of the value of application of Quality by Design (QbD) principles to analytical methods (Analytical Quality by Design; AQbD), including mass spectrometry, for procedural development and optimization^1,2^. The multi-dimensional combination and interaction of critical mass spectrometric variables constitutes a design space, which can be described in terms of multivariate mathematical modeling and characterized through application of a screening design of experiments (DOE)^3–5^. A regression model-based design space can be defined by a process involving selection of DOE, performance of randomized experiments, and data analysis^6^. Response surface methodology comprises a group of statistical techniques for empirical model building and model exploitation. By careful design and analysis of experiments, it can relate a response (output variable) to the number of predictors (input variables) that affect it^7,8^. A Box-Wilson central composite design, commonly referred to as central composite design (CCD), is an effective tool for construction of a second-order polynomial for the response variable in response surface methodology^9^, without implementation of a complete full factorial design of experiments. There is compelling evidence that CCD effectively improves LC-MS sensitivity and detection^10–12^.

The amino acid glutamine (Gln) is critical for optimal neurological function as it is processed to yield excitatory (glutamate, aspartate) and inhibitory (γ-amino butyric acid) amino acid neurotransmitters, and is an alternative source of energy during glucose deficiency^13^. The structural heterogeneity of the brain necessitates capabilities for accurate quantification of Gln in small volume brain tissue samples. In order to avoid disadvantages of available colorimetric, amperometric, and fluorescence Gln detection methods^14–19^, including issues arising from matrix interference, prolonged analysis duration, and analyte instability, we developed a combinatory high-resolution micropunch dissection/UHPLC-electrospray ionization mass spectrometric (LC-ESI-MS) approach for quantification of the fluorenylmethyloxycarbonyl (FMOC) derivative of Gln, e.g. Gln-FMOC in discrete brain structures. FMOC was used as a derivatizing agent as it is superior to other compounds such as benzoyl chloride and dansyl chloride for amine detection^20–22^. The initial phase of this research utilized CCD methodology, involving performance of DOE-recommended experiments, DOE-generated quadratic equation-based validation of predicted responses, and statistical comparison of design versus desirability in brain tissue samples, to assess five critical mass spectrometric process variables, e.g. sheath gas pressure (SGP), auxiliary gas pressure (AGP), sweep gas pressure (SWGP), ion transfer tube temperature (ITT), and vaporizer temperature (VT), on Gln-FMOC chromatographic area. CCD methodology was next employed to evaluate effects of critical liquid chromatographic process variables, such as column temperature, mobile phase flow rate, and mobile phase composition, in order to further optimization of analytical sensitivity for Gln quantification in neural tissue.

## Materials and methods

### Materials

LC-MS grade methanol was purchased from ThermoFisherScientific, Waltham, MA. Ammonium acetate was obtained from J.T. Baker, Radnor, PA. Sodium bicarbonate was purchased from Spectrum Chemicals Mfg. Corp, New Brunswick, NJ. 1-Adamantanamine hydrochloride 99% (AD), 9-fluorenylmethyl chloroformate 98+% (FMOC), and L-glutamine 99% (Gln) were obtained from Alfa Aesar, Haverhill, MA. Inserts, 350 µL, small volume, flat bottom, borosilicate glass, 6 × 31 mm, AQ Brand were purchased from Microsolv Technology Corporation, Leland, NC. National C5000-1W 2 mL Clear Glass ID Surestop vials was purchased from ThermoFisherSci.

### Pre-column FMOC derivatization of Gln

**Figure 1:**
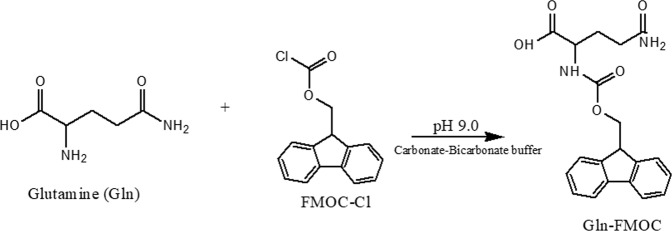
Glutamine (Gln) reacts with FMOC-Cl in carbonate buffer pH 9.0 to yield Gln-FMOC, which is detected at m/z [M-H] 367.1 in negative mode.

Procedures were adapted with minor modification from Rebane *et al*.^23^. A 0.4 mg/mL Gln stock solution was prepared in a 1.5 mL plastic microcentrifuge tube with ultrapure water. Separately, FMOC (1.5 mg) was dissolved in acetonitrile (1 mL), then vortexed to yield a clear solution, and AD (1 mg) was dissolved in 50% acetonitrile (1 mL). Accurately weighed sodium carbonate (10 mg) was transferred to ultrapure water (200 mL); after stirring to produce a clear solution, sodium bicarbonate solids (80–100 mg) were added under magnetic stirring to pH 9.0. Gln reaction with FMOC was initiated by combining Gln stock (100 µL), carbonate buffer pH 9.0 (100 µL), FMOC (100 µL); the mixture was vortexed (30 s) and allowed to stand at 25 °C (40 min). After addition of AD (50 µL), contents were vortexed (30 s), allowed to stand at 25 °C (5 min), and centrifuged to yield a clear supernatant, which was transferred to 350 µL inserts, which were transferred to 2 mL Surestop vials placed in an autosampler tray. The reaction is illustrated in Fig. 1.

### Liquid chromatography-mass spectrometry parameters

Samples analysis was performed using a system comprised of a UHPLC Vanquish pump, Vanquish Autosampler, Vanquish UHPLC + column compartment, and ISQ EC mass spectrometer (ThermoFisherSci.), in conjunction with ThermoFisherScientificDionexChromeleon 7 Chromatography Data System software. A C18 column (4.6 mm ID X 100 mm L, 5 µm, 120 Å; Acclaim 120; ThermoFisherSci.) was used with a 0.25 mL/min. flow rate and 0.5 µL injection volume; the autosampler needle was washed with 10% (v/v) methanol (10 s) after each sample injection. Mobile phases A and B consisted of 10 mM ammonium acetate and LC-MS grade acetonitrile, respectively. The linear gradient mobile phase flow was characterized by an increase in acetonitrile from 50% to 80% between 0 to 4 min, followed by a decrease to 50% between 4 to 8 or 15 min. Preliminary results guided selection of the following mass spectrometric parameters: vaporizer temperature (VT) = 250 °C; ion transfer tube temperature (ITT) = 200 °C; optimum sheath gas pressure (SGP) = 25 psig; auxiliary gas pressure (AGP) = 2 psig; sweep gas pressure (SWGP) = 0.5 psig; and negative mode, which resulted in an extracted Gln-FMOC chromatogram at m/z 367.1. Column and autosampler temperatures were maintained at 35 °C and 15 °C, respectively. This method represented here as Gen-MS-I.

### Rat brain tissue procurement for LC-ESI-MS measurement of glutamine

Adult male Sprague-Dawley rats (3–4 months of age) were handled in accordance with NIH guidelines for care and use of laboratory animals, under ULM Institutional Animal Care and Use Committee approval. Animals were sacrificed by microwave fixation. (1.45 sec exposure; *In Vivo* Microwave Fixation System, 5 kW; Stoelting Co., Wood Dale, IL) for optimal preservation of brain tissue. Individual brains were cut into serial 150 micron-thick frozen coronal sections. Random cortical neural tissue samples were obtained from sections using 1.50 mm calibrated hollow punches (Stoelting, Inc.), and collected into 100 µL of ultrapure water for storage at −80 °C.

### CCD design, modeling, and statistical analysis

**Figure 2:**
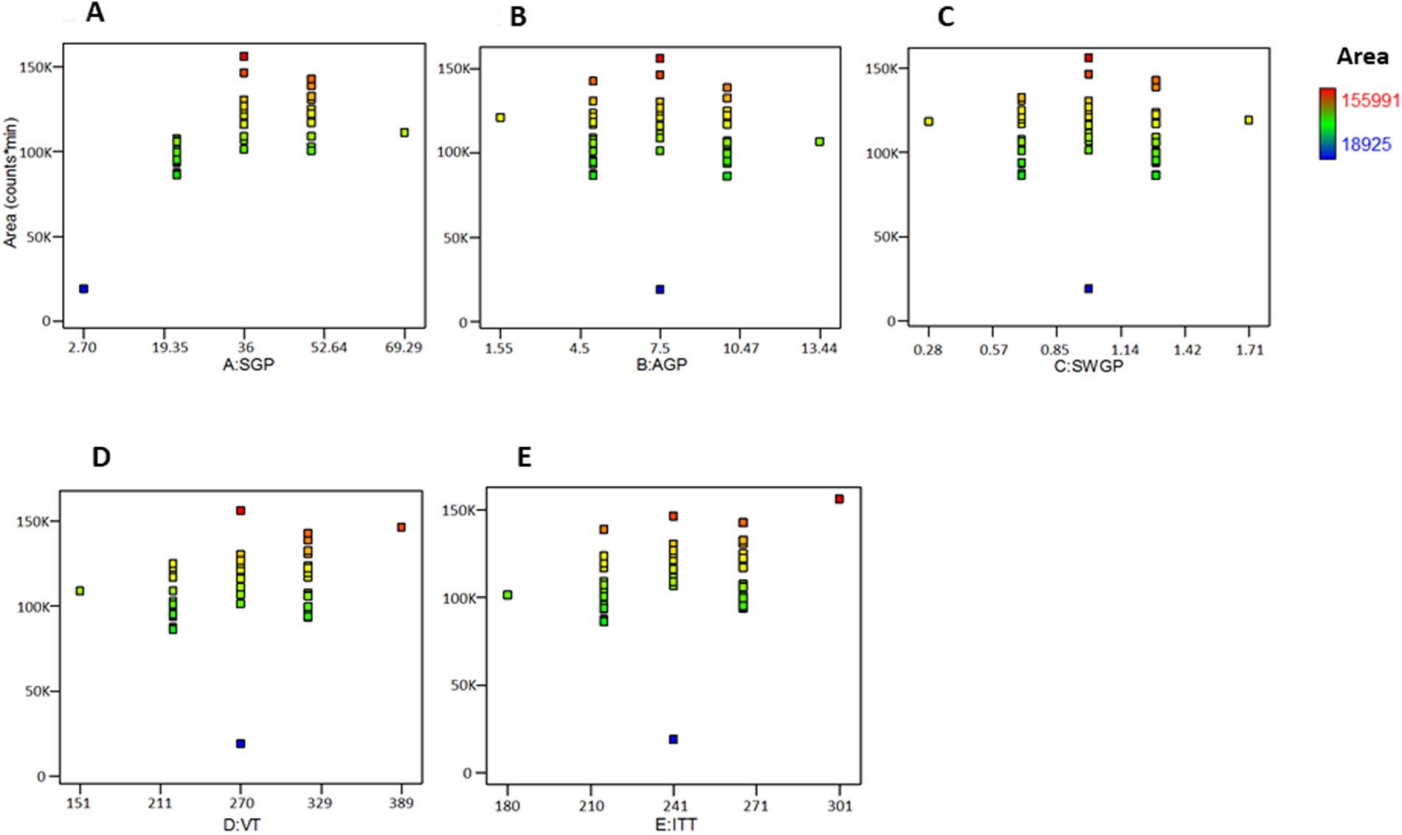
Mass spectrometric parameters optimization: Effects of SGP (psig), AGP (psig), SWGP (psig), VT (°C), or ITT (°C) on Gln-FMOC chromatographic area. SGP = 36 from panel A, AGP = 7.5 from panel B, SWGP = 1 from panel C, VT = 270 from panel D, and ITT = 301 from panel E produced maximum chromatographic response of standard Gln-FMOC.

VT, ITT, SGP, AGP, and SWGP were selected for optimization as independent and interactive mass spectrometric variables (Fig. 2) impacting the response variable, e.g. Gln-FMOC extracted chromatogram area-under-the-curve. Minimum and maximum values of each parameter (Supplementary Data; Table 1), chosen to meet minimum and maximum safe operative limits of the instrument, were utilized by Design Expert (Version: 9.0.6.2, Serial Number: 1997-5434-3543-9229) to create a series of 50 experimental runs for LC-ESI-MS analysis of Gln-FMOC (Suppl. Data; Table 2). Gln-FMOC chromatograph area measurements were analyzed by software to yield plot data, quadratic equation, and statistical results (Suppl. Data; Table 3). CCD methodology was similarly employed to assess effects of liquid chromatography column temperature, mobile phase flow rate, and mobile phase % acetonitrile (% B)−3min., % B-4.1 min., and % B-6 min. on Gln-FMOC chromatographic area, and to determine if and how optimization of these additional variables affects mass spectrometry performance.

## Results and Discussion

### Mass spectrometric optimization

Figure 2 depicts SGP (Panel A), AGP (Panel B), SWGP (Panel C), VT (Panel D), and ITT (Panel E) effects on chromatograph area. The plot of Normal % Probability vs. internally studentized residuals showed two outliers, indicating that most responses show normal distribution (Supp. Data Fig. 1A). Plots of predicted vs. internally studentized residuals and run number vs. internally studentized residuals showed random residual distribution around the horizontal axis, with one outlier violation, demonstrating nonlinear dependency between residuals and fitted values and therefore suitability of a regression model or non-linear model for data analysis (Supp. Data Fig. 1B,C). Plots of predicted vs. actual (area in counts) exhibited one experimental outlier (Suppl. Data, Fig. 2A). Cook’s distance (D_i_) was used to identify experimental runs (runs 13 and 46) that do not correspond to others (Suppl. Data 2B). The current model yielded leverage measures of less than 0.6, with design points distributed in three different unique leverage lanes (Suppl. Data, Fig. 2C). Very few experimental runs produced a DFFITS value that exceeded 1.0 (Suppl Data, Fig. 2D). Software analysis recommended a quadratic versus linear or cubic model, based on Sequential Model Sum of Squares [Type I], Lack of Fit Tests, and Model Summary Statistics (Suppl. Data, Table 4). ANOVA outcomes (Suppl. Data, Table 5) reveal that factors SGP, VT, ITT, and SGP have a significant impact on area. Lack-of-Fit F-value (=2.138942) denotes statistical insignificance.

**Figure 3:**
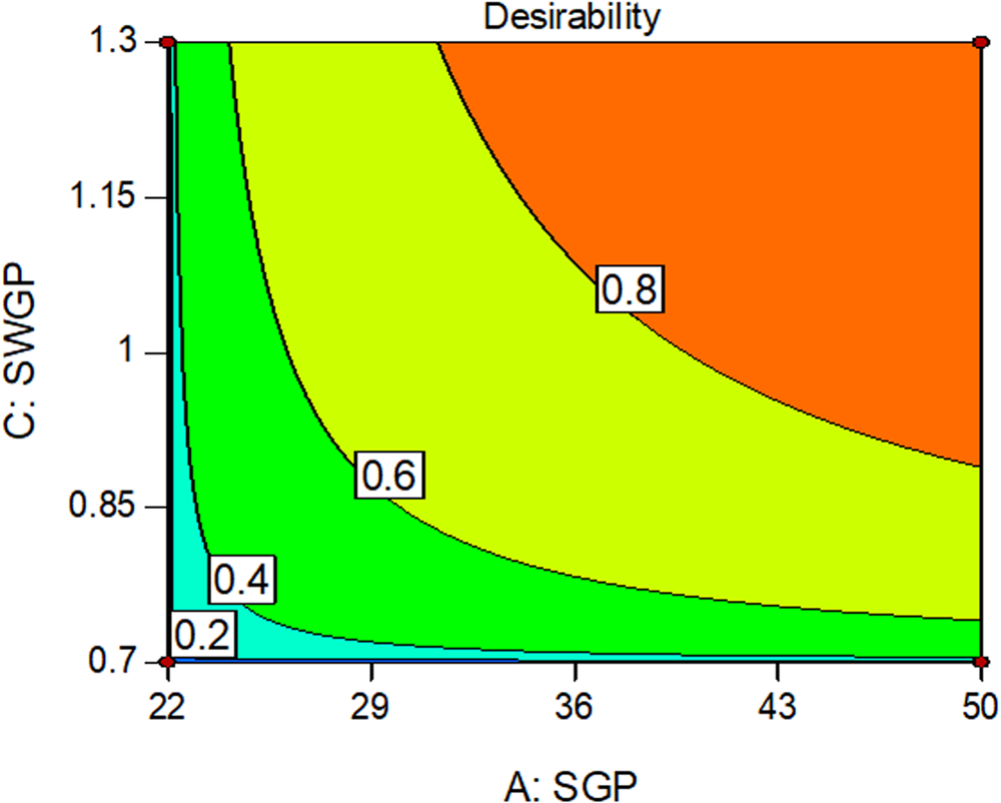
Contour plot of optimization through Desirability (0.975); SGP = 50, AGP = 10, SWGP = 1.3, VT = 320, ITT = 266 would produce a maximum response of standard Gln-FMOC.

**Figure 4:**
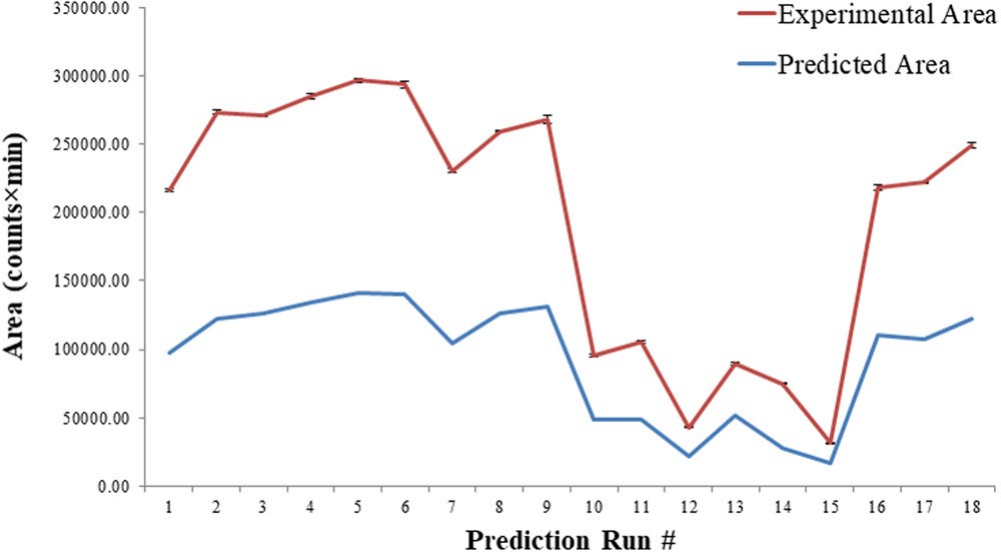
Areas for each run having a specific gas and temperature parameter. The predicted area obtained based on software generated quadratic equation, and experimental area was related to the implemented factor value in LC-ESI-MS. (n = 3), for Experimental Area in the freshly prepared Gln-FMOC for run). (*Refer* Supp. Data Table 6).

**Figure 5:**
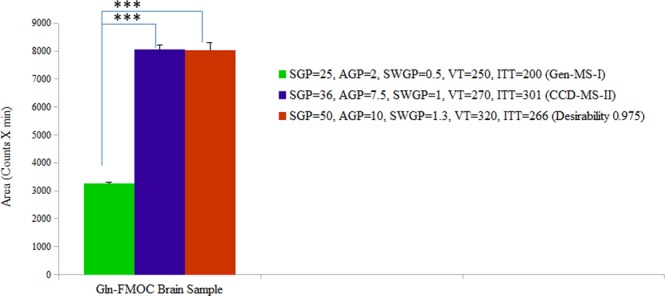
Comparison of Gln-FMOC response with parameters of preliminary general mass spectrometric response (Gen-MS-I), Central Composite Design-Mass Spectrometric optimization (CCD-MS-II), and Desirability Parameters in microwaved 150 µm thick micropunched cortical neural tissue(n = 3); ***P < 0.0001.

**Figure 6:**
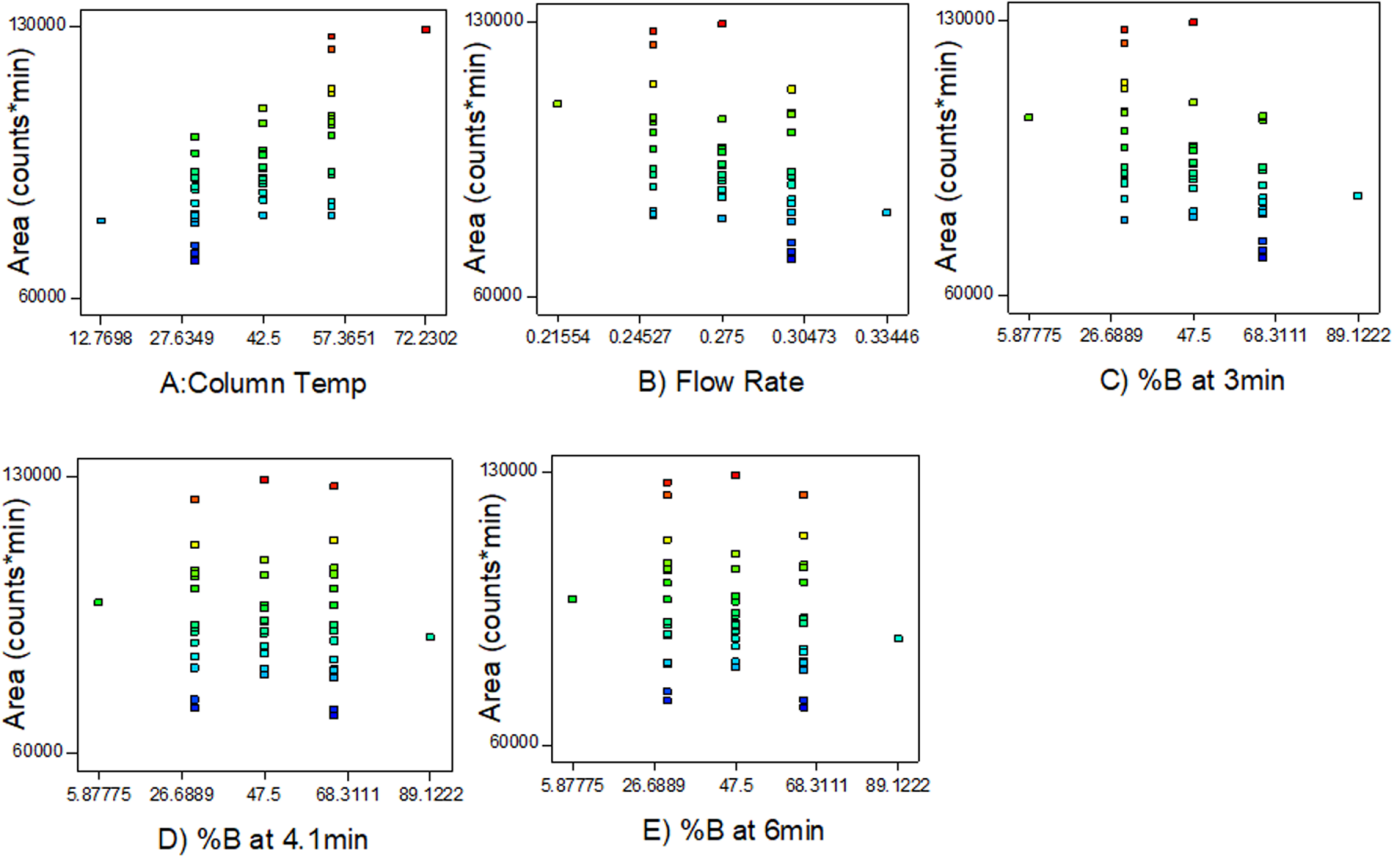
Liquid Chromatographic parameters optimization: Effects of column temperature (°C), flow rate (mL/min), % acetonitrile at 3-, 4.1-, 6-min respectively. Column temperature = 72.23 from panel 6 A, flow rate = 0.275 mL/min, % acetonitrile at 3-, 4.1-, 6-min = 47.5% showed maximum response of standard Gln-FMOC. (**A**) Effect of column temperature (°C) on chromatographic response of Gln-FMOC. (**B**) Effect of flow rate (ml/min) on chromatographic response of Gln-FMOC. (**C**) Effect of acetonitrile concentration (%B, v/v) at 3 min on chromatographic response of Gln-FMOC. (**D**) Effect of acetonitrile concentration (%B, v/v) at 4.1 min on chromatographic response of Gln-FMOC. (**E**) Effect of acetonitrile concentration (%B, v/v) at 6 min on chromatographic response of Gln-FMOC.

Analysis of combinatory mass spectrometric parameter effects on chromatographic area shows that this response was increased at medium AGP or SWGP levels, and decreased at higher AGP or SWGP levels under SGP influence (Suppl. Data, Fig. 3A,B). Improved chromatographic area occurred as a result of augmentation of either VT or ITT alongside increased SGP (Suppl. Data, Fig. 3C,D). Maximum chromatographic response was observed at medium AGP levels in combination with medium SWGP and higher VT and ITT levels, respectively (Suppl. Data, Fig. 4A–C). Medium SWGP and higher VT or ITT increased chromatographic response (Suppl. Data, Fig. 5A,B). Greatest chromatographic response was observed at maximum VT or ITT (Suppl. Data, Fig. 6). A maximum response of design can be achieved through desirability (97.5%), indicative of higher SGP = 50, and SWGP = 1.3 (Fig. 3). These data indicate that chromatographic response is maximized by increasing VT or ITT alongside SGP.

The software-generated quadratic equation, Response = −135346 + 3330.88826 (SGP) + 10537.4661 (AGP) + 51435.2088 (SWGP) + 155.82485 (VT) + 367.54438 (ITT) − 3.75179 (SGP × AGP) + 340.83333 (SGP × SWGP) + 2.39 (SGP × VT) + 2.48057 (SGP × ITT) − 1435.91667 (AGP × SWGP) + 4.462 (AGP × VT) − 24.35294 (AGP × ITT) + 43.44583 (SWGP × VT) − 165.62908 (SWGP × ITT) − 1.14235 (VT × ITT) − 54.09135 (SGP^2^) − 314.77362 (AGP^2^) − 12569.6642 (SWGP^2^) + 0.173494 (VT^2^) + 0.968247 (ITT^2^), identified synergistic factors that improve area (indicated by a positive sign) and antagonistic factors that decrease area (denoted by a negative sign). The equation shows that the constant, −135346, is independent of any single factor or factor interactions. The parameters SGP × AGP, AGP × SWGP, AGP × ITT, SWGP × ITT, VT × ITT, SGP^2^, AGP^2^, and SWGP^2^ decreased chromatographic area, whereas SGP, AGP, SWGP, VT, ITT, SGP × SWGP, SGP × VT, SGP × ITT, AGP × VT, SWGP × VT, VT^2^, and ITT^2^ enhanced this response.

### Fresh Gln-FMOC matching stock exhibited large differences in chromatographic response versus prediction analyses

**Figure 7:**
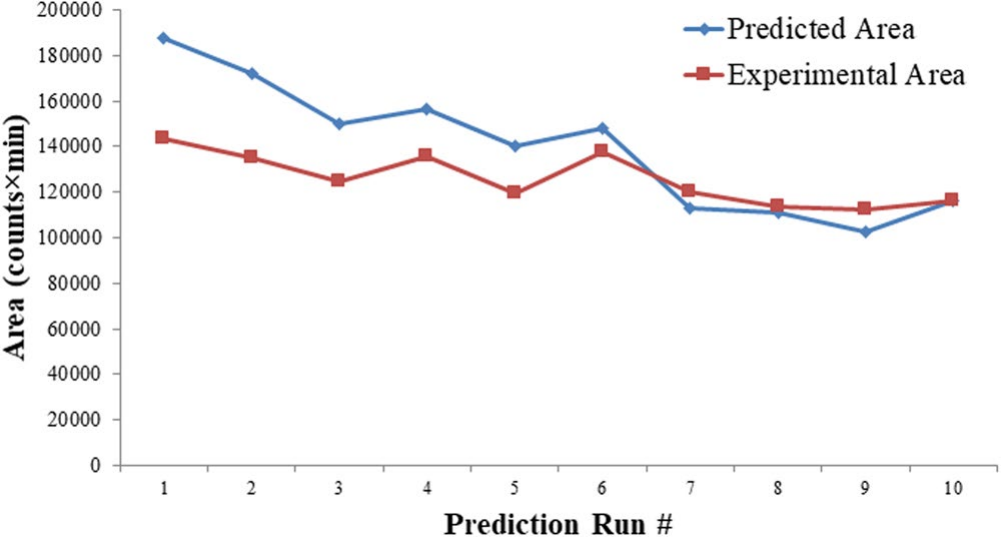
Area for each run having a specific column temp, flow rate, and %B at 3, 4.1, and 6 min. respectively. The predicted area was obtained based on software-generated quadratic equation, and experimental area was related to the implemented factor value in LC-ESI-MS. (n = 3, for Experimental Area in the freshly prepared Gln-FMOC) (*Refer* Supp. Data Table 12).

A matching concentration of Gln-FMOC was prepared for comparison of chromatographic response with design-predicted responses. Differences in response were approximately 3000 counts × min (Suppl Data, Fig. 7). The matching stock was used to evaluate chromatographic response under mass spectrometric parameter variation (Suppl Data, Table 6). Predictions of overlap between predicted versus experimental values were verified, but overlap was accompanied by differences in response area (Fig. 4; Suppl. Data Table 6).

#### Selection of highest mass spectrometric area based on design runs and desirability approach

Mass spectrometric optimization was performed using the above-described CCD technique, resulting in selection of maximum response parameters as VT = 270 °C, ITT = 301 °C, SGP = 36 °C, AGP = 7.5 °C, and SWGP = 1 °C (Design run #13 in parameters and response in Suppl. Data, Tables 2 and 3); optimized Gln-FMOC extracted mass chromatogram area-under-the-curve is denoted as CCD-MS-II. Cortical neural tissue samples were analyzed for Gln-FMOC response by utilizing Gen-MS-I, CCD-MS-II and desirability parameters and compared their difference in responses (Fig. 5). There was a significant difference between Gen-MS-I and CCD-MS-II, and Gen-MS-I and Desirability (P < 0.0001), but there was no significant difference between CCD-MS-II and Desirability. These observations led to selection of CCD-MS-II owing to lesser SGP, AGP, SWGP and VT over Desirability parameters, i.e. reduction in temperature requirement. Parameters of CCD-MS-II were kept constant during subsequent optimization of LC parameters toward further improvement of Gln-FMOC chromatographic response.

### Liquid chromatography optimization

DOE was used to evaluate effects of selected independent chromatography variables, e.g. column temperature, flow rate, % acetonitrile (% B)-3min., % B-4.1 min., and % B-6 min. on extracted mass chromatographic Gln-FMOC area-under-the-curve at [M-H] m/z 367.1. The low and high values of each parameter (Supplementary Data; Table 7), were utilized by Design Expert to create a series of 50 experimental runs for LC-ESI-MS analysis of Gln-FMOC (Suppl. Data; Table 8), which were performed to obtain response (Suppl. Data, Table 9). Gln-FMOC chromatograph area measurements were analyzed by software to produce plot data, quadratic equation, and statistical results (Suppl. Data, Tables 10 and 11).

**Figure 8:**
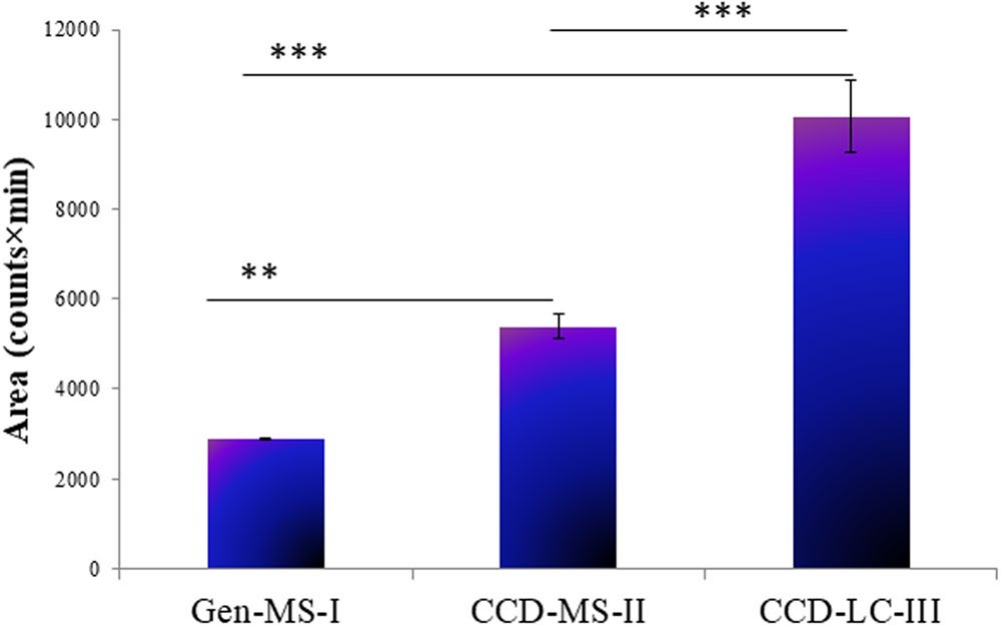
Comparison of Gln-FMOC response with parameters of preliminary general mass spectrometric response (Gen-MS-I), Central Composite Design-Mass Spectrometric optimization (CCD-MS-II), and Central Composite Design-Liquid Chromatography (CCD-LC-III) parameters in microwaved 150 µm thick micropunched cortical neural tissue. CCD-MS-II optimization improved the sensitivity by 1.8 times over Gen-MS-I. CCD-LC-III optimization further enhanced the response by 3.5 times over Gen-MS-I, and 1.8 times over CCD-MS-II(n = 3). ***P < 0.0001 and **P < 0.001.

**Figure 9:**
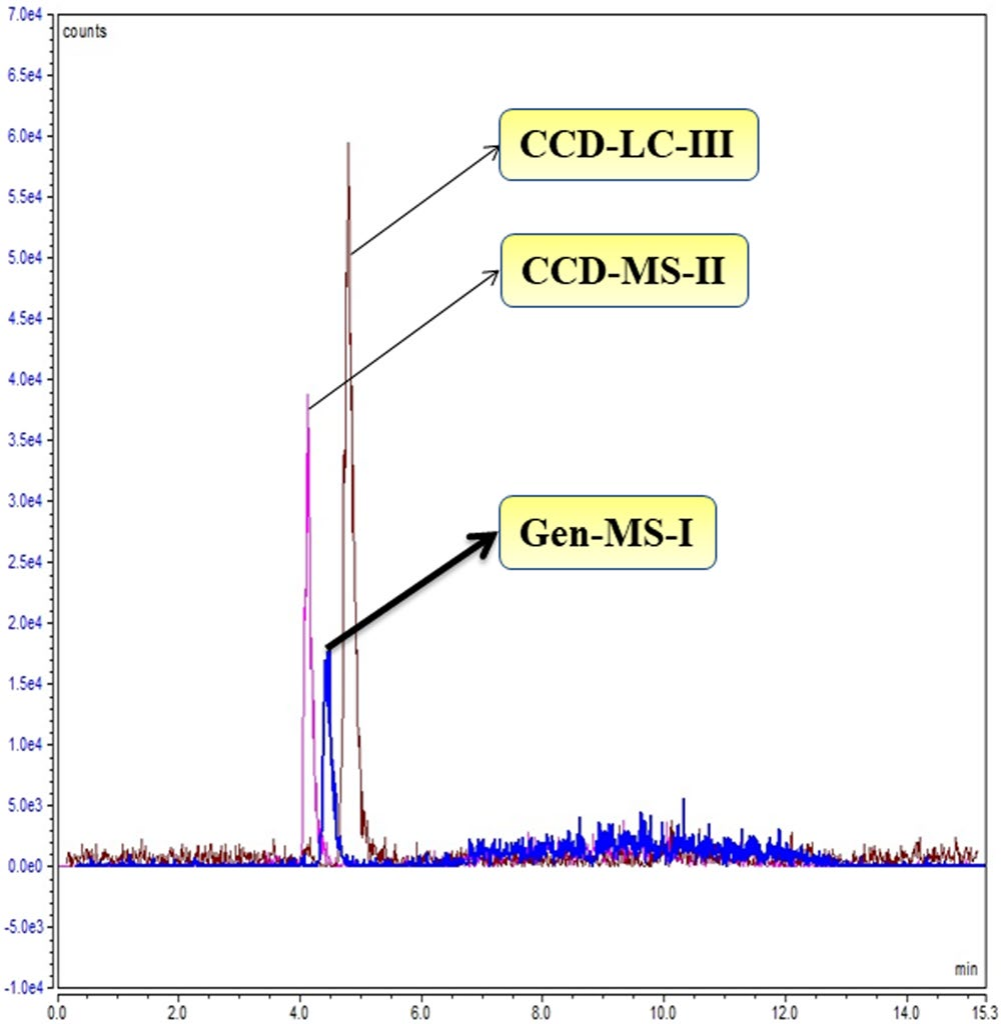
Extracted overlay chromatogram of Gln-FMOC at [M-H] m/z 367.1 from microwaved 150 µm thick micropunched cortical neural tissue through Gen-MS-I, CCD-MS-II, and CCD-LC-III parameters. CCD-LC-III chromatogram has the highest area over CCD-MS-II and Gen-MS-I.

Figure 6A–E contain graphical representations of column temperature, flow rate, % B-3min., % B-4.1 min., and % B-6 min. versus chromatograph area. ANOVA outcomes show that the individual factors column temperature, flow rate, %B-3min. %B-4.1 min, and %B-6 min; combined factors of column temperature and %B-3min; and square of the column temperature significantly impact area (Suppl. Data, Table 11). There was one outlier violation in normal percent probability plot, internally studentized residuals (Suppl. Data, Fig. 8). No outlier in plots of predicted vs. actual (area in counts) (Suppl. Data, Fig. 9A), Cook’s distance (D_i_) was less than 0.8 (Suppl. Data 9B), the design points three distinctive leverage lanes (Suppl. Data, Fig. 9C), and most experimental runs were in the control limits of DFFITS (Suppl Data, Fig. 9D). Contour plots of combinatory effects of liquid chromatography parameters on chromatograph area are shown in Suppl. Data Figs. 10–13.

Software generated the following quadratic equation: Response = 167794 + 1399.26988 (Column temperature) − 430278 (Flow rate) − 114.20386 (%B at 3 min) + 15.45278 (%B at 4.1 min) − 421.86548 (%B at 6 min) − 4347.9 (Column temperature × Flow rate) − 8.55757 (Column temperature × %B at 3 min) − 2.16967 (Column temperature × %B at 4.1 min) − 0.149667 (Column temperature × %B at 6 min) − 709.69048 (Flow rate × %B at 3 min) − 57.7381 (Flow rate × %B at 4.1 min) + 795.69048 (Flow rate × %B at 6 min) − 0.518401 (%B at 3 min. × %B at 4.1 min) + 3.04133 (%B at 3 min. × %B at 6 min) − 0.962959 (%B at 4.1 min. × %B at 6 min) + 13.27048 (Column temperature)^2^ + 646217 (Flow rate)^2^ + 1.33141 (%B at 3 min)^2^ + 0.501067 (%B at 4.1 min)^2^ – 0.309845 (%B at 6 min)^2^. The constant, 167794, is independent of any single factor or factor interaction. Parameters decreasing chromatographic area were identified as %B-3min., %B-6min., Column temperature × Flow rate, Column temperature × %B-3min., Column temperature × %B-4.1 min., Column temperature × %B-6min, Flow rate × %B-3min., Flow rate × %B-4.1 min., %B-3min. × %B-4.1 min., %B-4.1 min. × %B-6min., and (%B at 6 min.)^2^. Synergistic effects were elicited by Column temperature, %B-4.1 min., Flow rate × %B-6min., %B-3min. × %B-min., (Column temperature)^2^, (Flow rate)^2^, (%B at 3 min.)^2^, and (%B at 4.1 min.)^2^.

Similar prediction study was performed for LC optimization, and the predicted responses pattern were in alignment with experimental response (Fig. 7; Supp. Data Table 12). LC parameters of Prediction run-4 were similar to the design parameters run 19 (Supp. Data Tables 8 and 12), which was compared with run-1 with a view to observe the response maximum between the design and prediction run. Prediction run-1 showed significantly maximum response when compared to prediction run 4 (P < 0.05) based on unpaired two tailed t-test (Supp. Data Fig. 14; Supp. Data Table 12). This indicates that quadratic equation is potential in producing the higher responses over the design set parameters. The Prediction run-1 parameters were denoted here as CCD-LC-III.

#### Comparison of MS and LC optimization for maximum chromatographic response

Improvement in analytical method sensitivity was verified by comparison of Gen-MS-I, CCD-MS-II, and CCD-LC-III parameters on the response variable. Microwaved cortical neural tissue was derivatized with FMOC for detection of Gln-FMOC at m/z 367.1, and chromatographic responses was compared between Gen-MS-I, CCD-MS-II, and CCD-LC-III parameters (Fig. 8). Data show that CCD-MS-II resulted in significantly higher area compared to Gen-MS-I; CCD-LC-III produced significantly greater chromatographic versus Gen-MS-I and CCD-MS-II. An overlay of extracted chromatograms of Gln-FMOC using these three methods at [M-H] m/z 367.1 in brain tissue is present in Fig. 9, Supp. Data Tables 13 and 14.

In summary, DOE-based evaluation of liquid chromatography parameters improved sensitivity of ESI-MS detection of Gln, thereby reducing sample volume requirements for precise quantification of this amino acid. As CCD augmentation of Gln-FMOC is selective for that derivative, similar optimization schemes will be required for other critical brain amino acids.

## Conclusions

In the current studies, application of CCD for optimization of mass spectrometric detection of Gln-FMOC revealed significant individual and interactive effects of the critical variables AGP, SGP, SWGP, VT, and ITT on chromatographic area. CCD significantly augmented Gln-FMOC response compared to original mass spectrometric parameters. This outcome affords higher analytical selectivity with least sample injection volume, which is beneficial for reduction in biological sample volume requirement for discriminative quantification of Gln. Efficiency of improvement of analytical sensitivity is indicated by equivalence of highest design response in the design with desirability parameters. Observations of differential yields of experimental response compared to area predicted by quadratic equation owing to matching stock of Gln supported the necessity of performance of Gln analyses on an ad-hoc basis.

CCD-LC-III optimization revealed that column temperature and mobile phase composition is indispensible for improving the mass chromatographic response over the optimization of CCD-MS-II alone. Quadratic equation allowed generating significantly higher chromatographic response when compared to design parameters in LC optimization. Higher acetonitrile concentration is essential at peak elevation besides higher column temperature; this maximum acetonitrile concentration is not essential at peak terminals. The present CCD-LC method favors further decrease in the brain tissue sample for analysis of Gln and brain microstructures.

## Supplementary information

Supplementary information.

## Data Availability

All data generated or analysed during this study are included in this published article (and its Supplementary Information files).
