# Supplementary material for: Optimization of Ultra-High-Performance Liquid Chromatography-Electrospray Ionization-Mass Spectrometry Detection of Glutamine-FMOC Ad-Hoc Derivative by Central Composite Design
Source: Sci Rep. 2020 Apr 28;10:7134. doi: 10.1038/s41598-020-64099-w (PMC7188808; doi:10.1038/s41598-020-64099-w)
Supplement: Supplementary file 1 — Supplementary information. [file 41598_2020_64099_MOESM1_ESM.pdf]

Supplementary Data

Tables 1 - 14

Figures 1- 14

Table 1.

Code	Variable	Low	High
A	SGP (psig)	22	50
B	AGP (psig)	5	10
C	SWGP (psig)	0.7	1.3
D	VT (ºC)	220	320
E	ITT (ºC)	215	266

Table 2.

Run	SGP	AGP	SWGP[2]	VT	ITT[1]
1	50	10	1.3	320	215
2	22	10	0.7	320	215
3	50	10	0.7	220	266
4	36	7.5	1	270	240.5
5	36	7.5	1	270	240.5
6	50	10	0.7	220	215
7	22	5	0.7	220	266
8	50	5	0.7	220	266
9	22	5	1.3	320	215
10	22	5	0.7	320	266
11	22	10	1.3	320	215
12	36	7.5	1	270	240.5
13	36	7.5	1	270	301.15
14	36	7.5	1	270	240.5
15	50	5	0.7	320	215
16	36	7.5	1	270	240.5
17	36	7.5	1.71352	270	240.5
18	36	7.5	1	270	179.85
19	22	5	1.3	320	266
20	50	10	1.3	220	215
21	22	5	1.3	220	215
22	22	10	1.3	320	266
23	50	5	1.3	220	266
24	2.7022	7.5	1	270	240.5
25	36	13.446	1	270	240.5
26	50	5	0.7	320	266
27	22	10	0.7	220	266
28	50	10	0.7	320	215
29	50	5	1.3	320	266
30	50	10	1.3	320	266
31	22	5	0.7	220	215
32	22	10	0.7	320	266
33	22	5	1.3	220	266
34	50	10	0.7	320	266
35	50	5	1.3	220	215
36	50	10	1.3	220	266
37	36	7.5	1	388.921	240.5
38	36	7.5	1	270	240.5
39	36	1.55396[3]	1	270	240.5
40	22	10	0.7	220	215
41	50	5	0.7	220	215
42	36	7.5	1	270	240.5
43	36	7.5	0.28648	270	240.5
44	36	7.5	1	151.079	240.5
45	22	5	0.7	320	215
46	69.2978	7.5	1	270	240.5
47	50	5	1.3	320	215
48	22	10	1.3	220	215
49	36	7.5	1	270	240.5
50	22	10	1.3	220	266

[1] Nearest integer selected; [2] First decimal value selected;
[3] input value 2 instead 1.55396 was recommended by LC-ESI-MS software.

Table 3.

Run	Response-I	Response-II	Response-III	Mean	RSD (%)
1	138688	138237	132962	136629	2.33
2	107162	105454	104124	105580	1.44
3	124797	124937	123837	124523.67	0.48
4	130171	129308	131364	130281	0.79
5	127741	131147	129105	129331	1.32
6	102457	105903	107444	105268	2.42
7	100877	102924	100528	101443	1.27
8	121175	124125	124425	123241.67	1.45
9	95359	95054	93897	94770	0.81
10	107369	104369	104225	105321	1.68
11	93723	100418	94442	96194.333	3.82
12	115925	120266	118910	118367	1.87
13	155991	156563	158249	156934.33	0.74
14	116680	119114	118790	118194.67	1.11
15	116896	121687	115038	117873.67	2.91
16	116075	119564	117352	117663.67	1.50
17	118974	118658	121261	119631	1.18
18	101134	99598	102397	101043	1.38
19	105640	100633	100959	102410.67	2.73
20	100312	99818	106727	102285.67	3.76
21	86382	85623	84928	85644.333	0.84
22	99579	102173	101419	101057	1.32
23	117967	113120	116168	115751.67	2.11
24	18925	17548	19495	18656	5.36
25	106692	105650	107959	106767	1.08

Run	Response-I	Response-II	Response-III	Mean	RSD (%)
26	130753	140994	135668	135805	3.77
27	93827	92452	98439	94906	3.30
28	119470	120294	121547	120437	0.86
29	142446	139344	138934	140241.33	1.36
30	122092	131584	134221	129299	4.93
31	87321	84661	85524	85835.333	1.58
32	106024	108568	106391	106994.33	1.28
33	94461	96672	93727	94953.333	1.61
34	132536	136580	127591	132235.67	3.40
35	108851	100619	105489	104986.33	3.94
36	116801	116172	113761	115578	1.38
37	146296	148590	144858	146581.33	1.28
38	120713	123016	121203	121644	0.99
39	121052	121722	121661	121478.33	0.30
40	86285	85612	84885	85594	0.81
41	102594	100364	102503	101820.33	1.23
42	124993	125894	123366	124751	1.02
43	118229	123890	121726	121281.67	2.35
44	108613	109143	107666	108474	0.68
45	93315	92871	93831	93339	0.51
46	111130	110703	112644	111492.33	0.91
47	123545	116864	124485	121631.33	3.41
48	86033	88335	86113	86827	1.50
49	126764	129238	126497	127499.67	1.18
50	95219	96726	98377	96774	1.63

Figure 1.

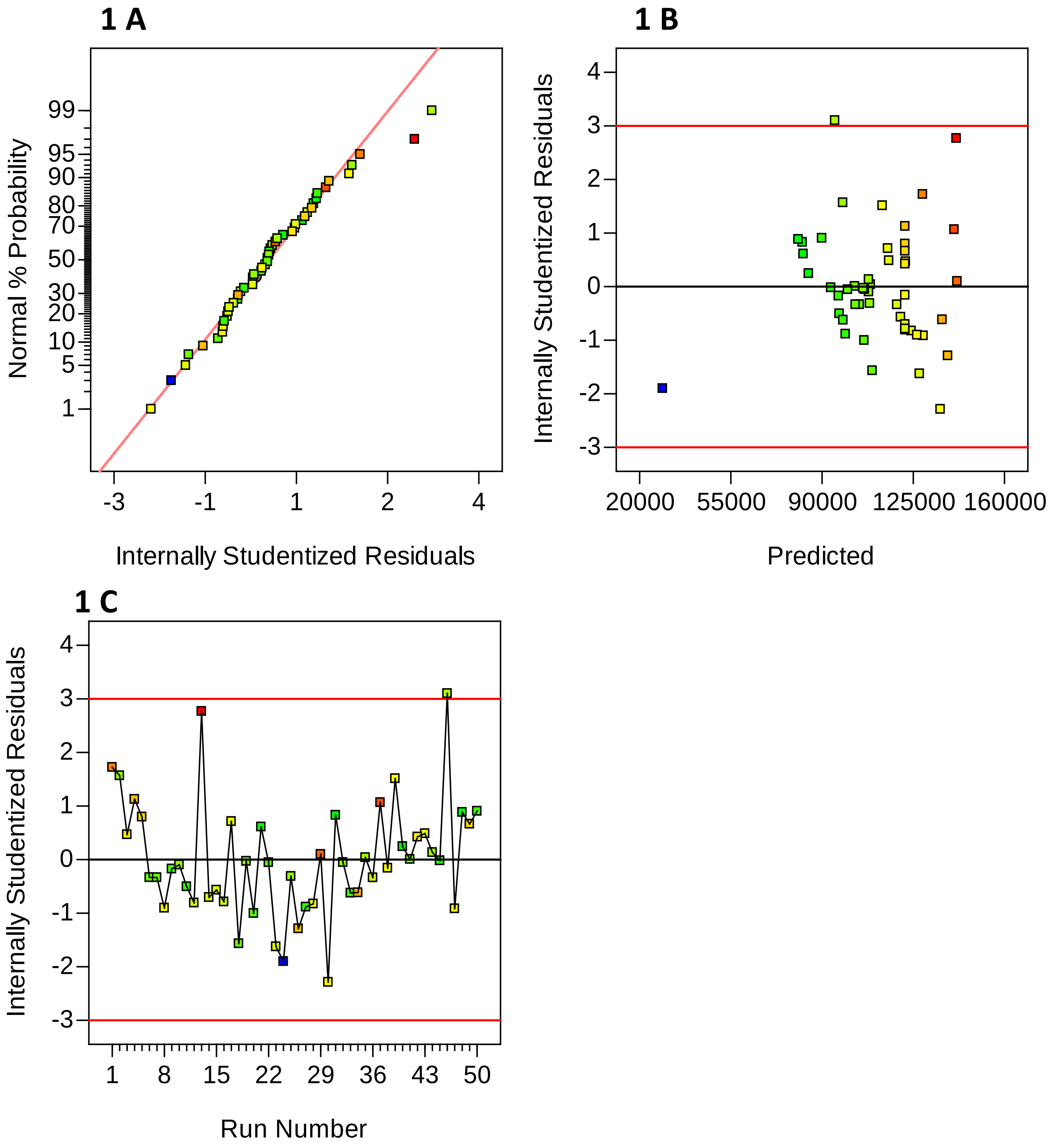
1 A
Normal % Probability
99
95
90
80
70
50
30
20
10
5
1
-3
-1
1
2
4
Internally Studentized Residuals
1 B
Internally Studentized Residuals
4
3
2
1
0
-1
-2
-3
20000
55000
90000
125000
160000
Predicted
1 C
Internally Studentized Residuals
4
3
2
1
0
-1
-2
-3
1
8
15
22
29
36
43
50
Run Number

Figure 2.

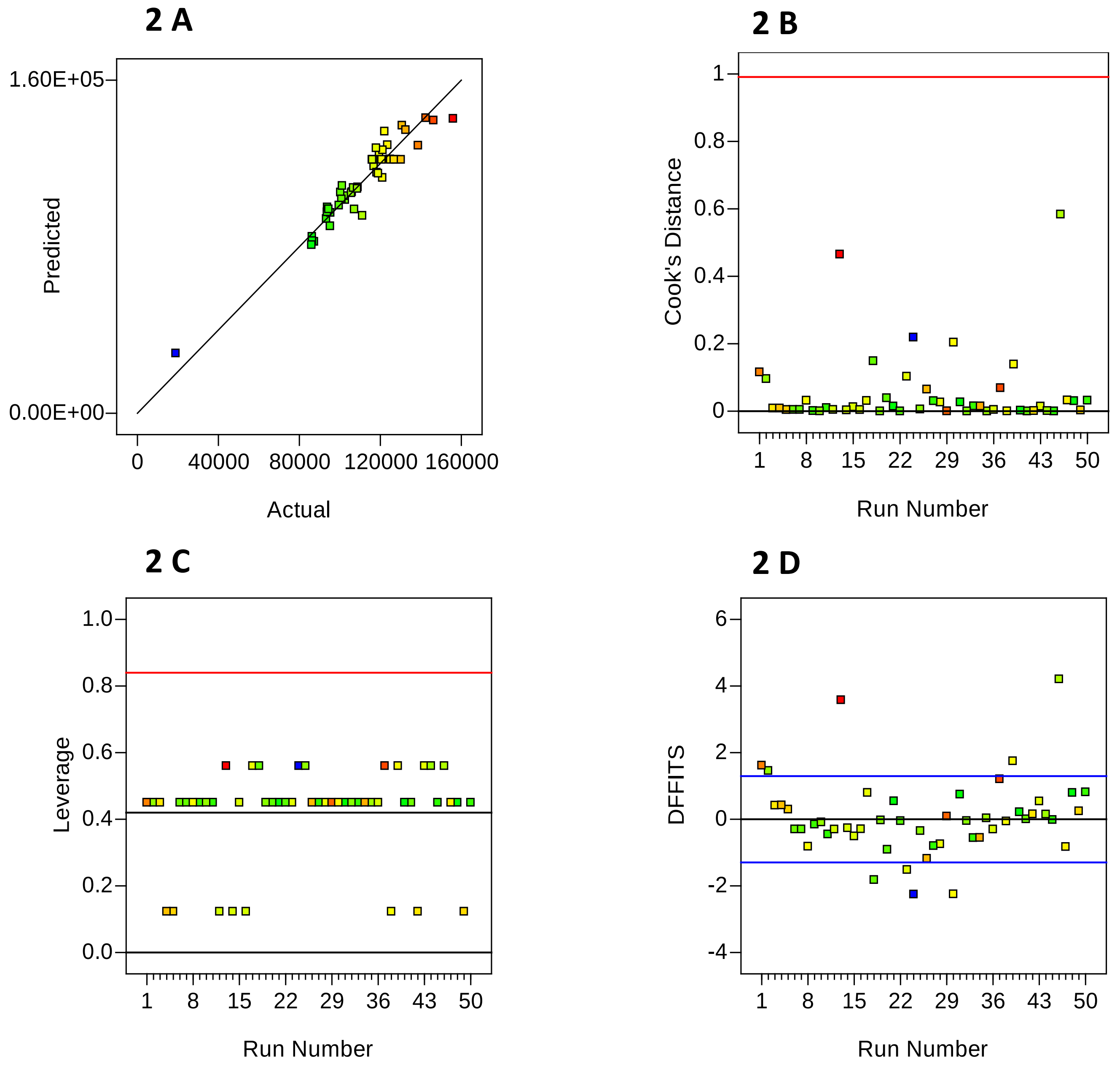
2 A
1.60E+05
0.00E+00
Predicted
0
40000
80000
120000
160000
Actual
2 B
1
0.8
0.6
0.4
0.2
0
Cook's Distance
1
8
15
22
29
36
43
50
Run Number
2 C
1.0
0.8
0.6
0.4
0.2
0.0
Leverage
1
8
15
22
29
36
43
50
Run Number
2 D
6
4
2
0
-2
-4
DFFITS
1
8
15
22
29
36
43
50
Run Number

Table 4.

A)

Source	Sequential p-value	Lack of Fit p-value	Adjusted R-Squared	Predicted R-Squared	
Linear	1.70959E-07	0.005869154	0.53584903	0.446291918	
2FI	0.996977414	0.002742207	0.42888809	0.381168838	
Quadratic	5.61767E-09	0.152542858	0.8582919	0.678961949	**Suggested**
Cubic	0.141081908	0.264045063	0.89952894	-0.283328963	Aliased

B)

Source	Sum of Squares	df	Mean Square	F Value	p-value Prob > F	
Mean vs Total	6.15174E+11	1	6.15174E+11			
Linear vs Mean	12468051551	5	2493610310	12.31381991	1.70959E-07	
2FI vs Linear	438401949.8	10	43840194.98	0.175944111	0.996977414	
Quadratic vs 2FI	6678864594	5	1335772919	21.60535643	5.61767E-09	**Suggested**
Cubic vs Quadratic	1179269154	15	78617943.58	1.793511523	0.141081908	Aliased
Residual	613685050.9	14	43834646.49			
Total	6.36553E+11	50	12731051434			

C)

Source	Sum of Squares	df	Mean Square	F Value	p-value (Prob > F)	
Linear	8678044643	37	234541747.1	7.071322978	0.005869154	
2FI	8239642693	27	305171951.6	9.200790308	0.002742207	
Quadratic	1560778099	22	70944459.05	2.138941956	0.152542858	**Suggested**
Cubic	381508945.4	7	54501277.91	1.643187806	0.264045063	Aliased
Pure Error	232176105.5	7	33168015.07			

D)

Source	Std.Dev.	R-Squared	Adjusted R-Squared	Predicted R-Squared	PRESS	
Linear	14230.42575	0.583211374	0.53584903	0.446291918	11837322146	
2FI	15785.15572	0.603718267	0.42888809	0.381168838	13229541099	
Quadratic	7862.951548	0.916131941	0.8582919	0.678961949	6863238881	**Suggested**
Cubic	6620.773859	0.971293983	0.89952894	-0.283328963	27435356026	Aliased

Table 5.

ANOVA for Response Surface Quadratic model						
Analysis of variance table [Partial sum of squares - Type III]						
Source	Sum of Squares	df	Mean Square	F value	p-value Prob > F	
Model	1.96E+10	20	9.79E+08	15.83906	1.23E-10	Significant
A-SGP	8.37E+09	1	8.37E+09	135.3782	1.91E-12	Significant
B-AGP	44900623	1	44900623	0.726242	0.401087	Not significant
C-SWGP	367200	1	367200	0.005939	0.9391	Not significant
D-VT	2.06E+09	1	2.06E+09	33.35416	2.96E-06	Significant
E-ITT	1.99E+09	1	1.99E+09	32.19895	3.9E-06	Significant
AB	551775.1	1	551775.1	0.008925	0.925385	Not significant
AC	65574152	1	65574152	1.060624	0.311586	Not significant
AD	90135451	1	90135451	1.457889	0.237022	Not significant
AE	25095070	1	25095070	0.405898	0.529057	Not significant
BC	37113420	1	37113420	0.600288	0.44474	Not significant
BD	9954722	1	9954722	0.161012	0.691168	Not significant
BE	77128200	1	77128200	1.247504	0.273198	Not significant
CD	13590291	1	13590291	0.219815	0.642686	Not significant
CE	51374316	1	51374316	0.83095	0.369514	Not significant
DE	67884552	1	67884552	1.097993	0.303362	Not significant
A^2	6.25E+09	1	6.25E+09	101.0249	5.88E-11	Significant
B^2	2.15E+08	1	2.15E+08	3.478703	0.072311	Not significant
C^2	71115500	1	71115500	1.150252	0.292341	Not significant
D^2	10453927	1	10453927	0.169086	0.683948	Not significant
E^2	22027506	1	22027506	0.356282	0.55521	Not significant
Residual	1.79E+09	29	61826007			
Lack of Fit	1.56E+09	22	70944459	2.138942	0.152543	Not significant
Pure Error	2.32E+08	7	33168015			

Figure 3.

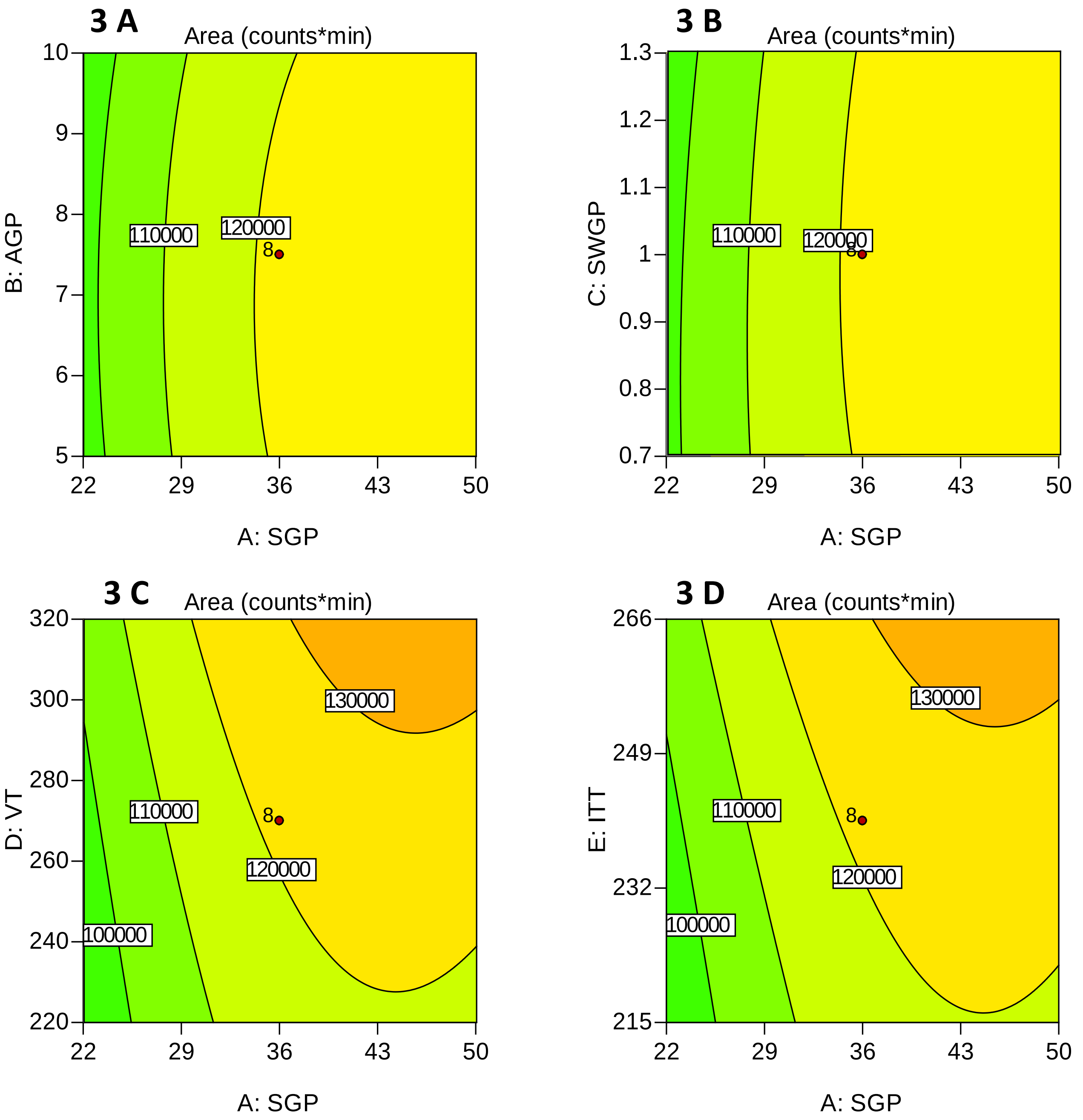
3 A
Area (counts*min)
B: AGP
A: SGP
110000
120000
8
3 B
Area (counts*min)
C: SWGP
A: SGP
110000
120000
8
3 C
Area (counts*min)
D: VT
A: SGP
100000
110000
120000
130000
8
3 D
Area (counts*min)
E: ITT
A: SGP
100000
110000
120000
130000
8

Figure 4.

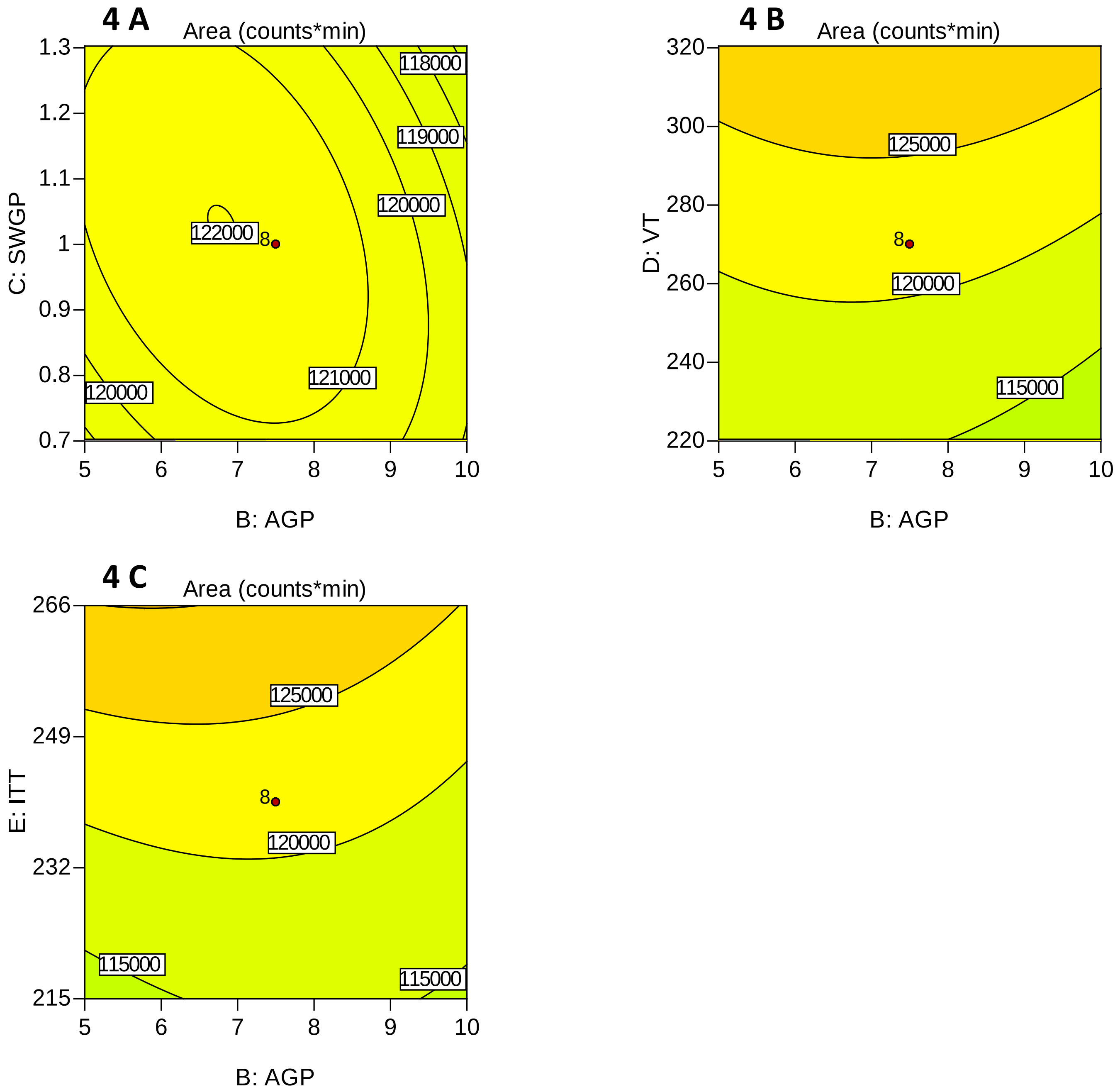
4 A
Area (counts*min)
C: SWGP
B: AGP
118000
119000
120000
122000
121000
120000
4 B
Area (counts*min)
D: VT
B: AGP
125000
120000
115000
4 C
Area (counts*min)
E: ITT
B: AGP
125000
120000
115000
115000

Figure 5.

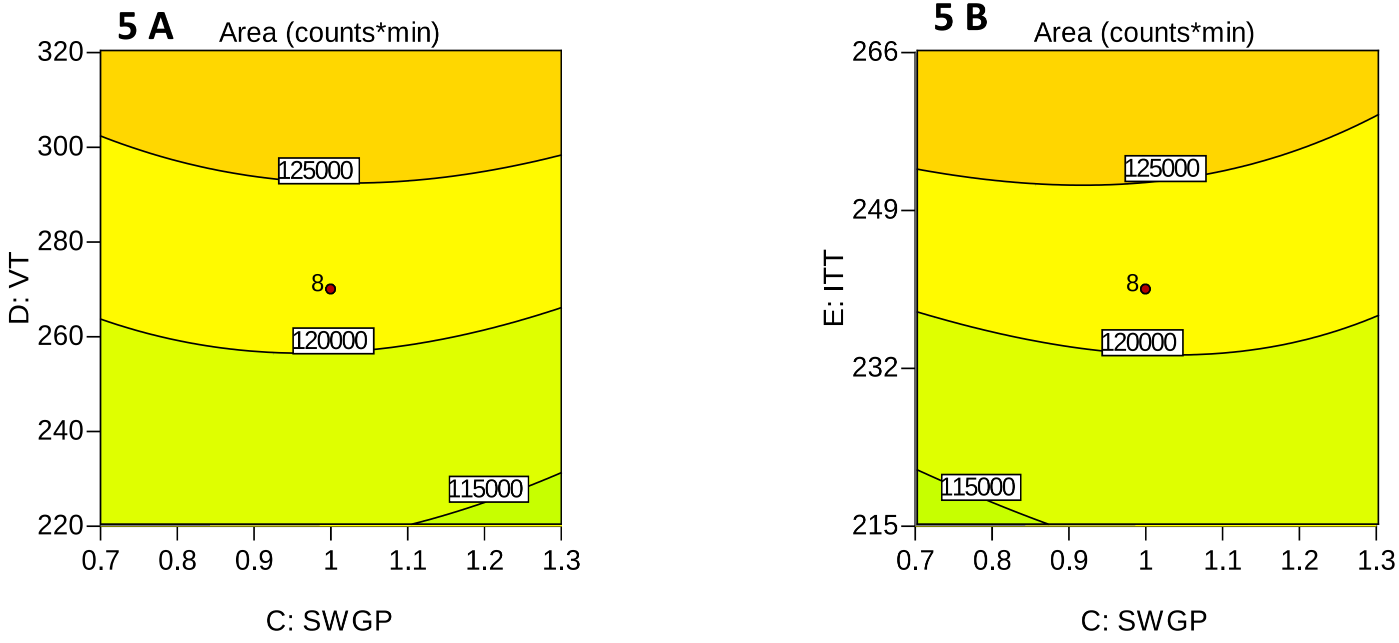
5 A
Area (counts*min)
D: VT
320
300
280
260
240
220
125000
120000
115000
8
0.7
0.8
0.9
1
1.1
1.2
1.3
C: SWGP
5 B
Area (counts*min)
E: ITT
266
249
232
215
125000
120000
115000
8
0.7
0.8
0.9
1
1.1
1.2
1.3
C: SWGP

Figure 6.

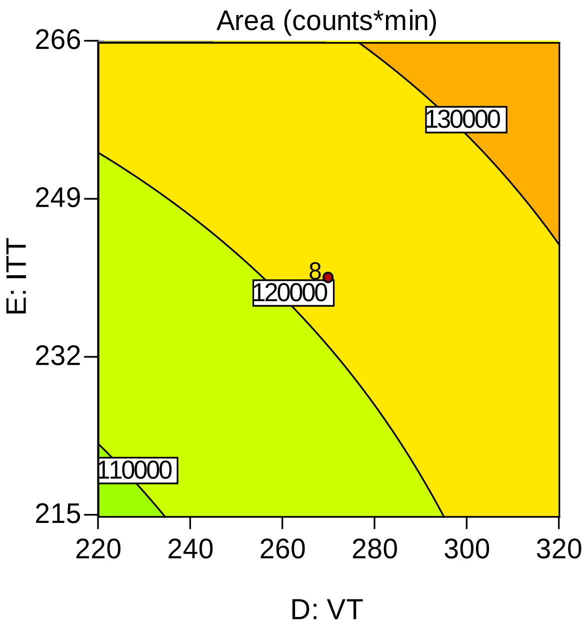

Area (counts*min)
266
249
232
215
E: ITT
130000
8
120000
110000
220
240
260
280
300
320
D: VT

Figure. 7

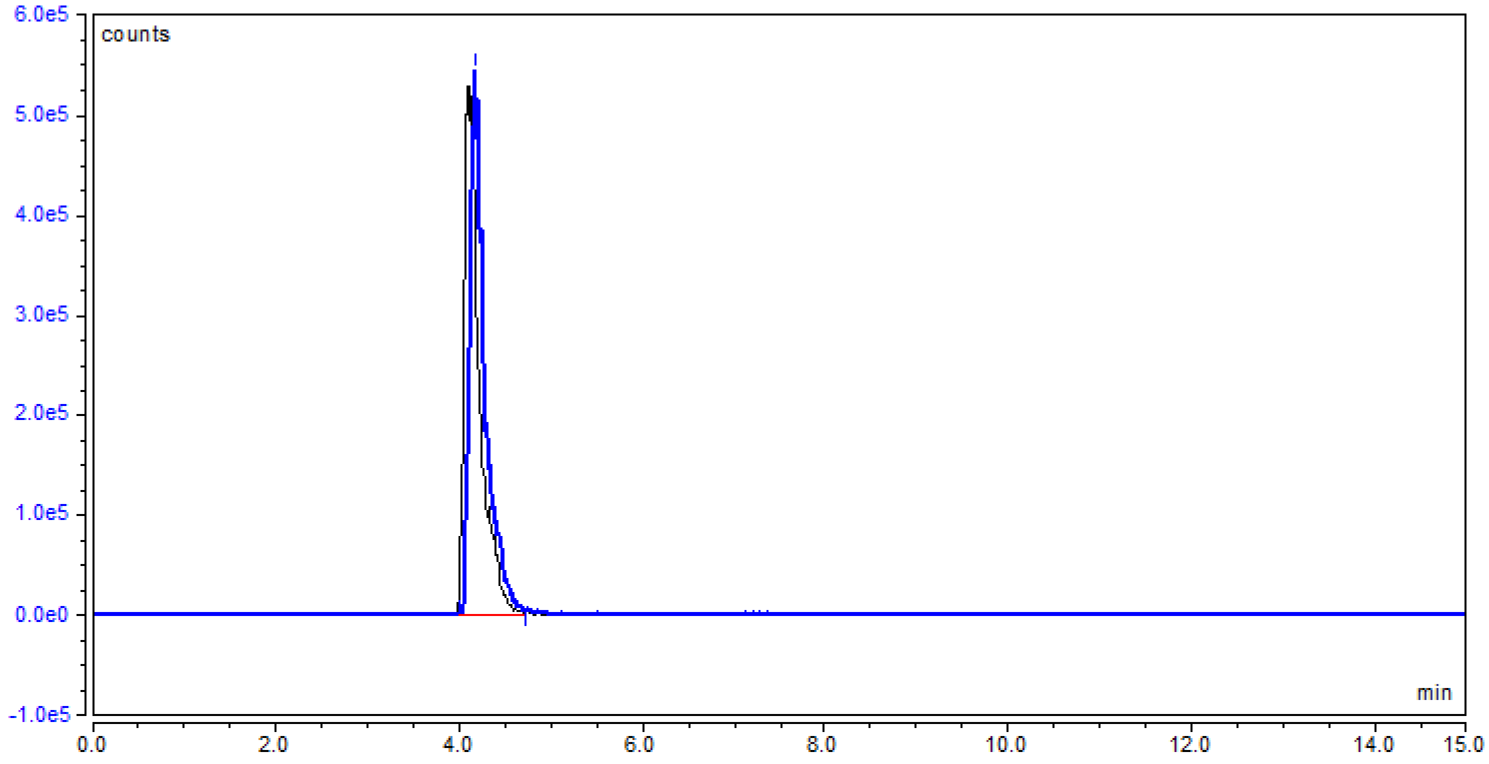
counts
6.0e5
5.0e5
4.0e5
3.0e5
2.0e5
1.0e5
0.0e0
-1.0e5
min
0.0
2.0
4.0
6.0
8.0
10.0
12.0
14.0
15.0

Table 6.

Prediction Run	SGP	AGP	SWGP	VT	ITT	Predicted Area	Experimental Area
1	22	7.5	1.006	272	242.54	98013.67	118584.67
2	36	7.5	1.006	272	242.54	122683.23	150743.67
3	50	7.5	1.006	272	242.54	126148.99	144819.67
4	50	7.5	1.006	320	242.54	134717.89	150587.33
5	50	7.5	1.006	320	266	141031.99	155813.67
6	50	7.5	1.3	320	266	140610.74	153123.00
7	25	7.5	1	270	240.5	104401.31	126092.67
8	45	7.5	1	270	240.5	126423.61	132912.67
9	45	7.5	1	300	240.5	131367.12	136567.00
10	10	5	0.5	220	220	48397.23	47715.33
11	10	5	0.2	240	240	48397.23	57027.67
12	5	5	0.5	200	200	21936.21	21332.33
13	7.5	5	0.5	270	250	51797.44	37862.00
14	10	10	2	235	200	28057.52	47108.50
15	2	5	0.4	245	220	17211.29	14213.00
16	30	10	0.7	260	240	110360.39	108005.00
17	45	5	0.3	280	220	107260.31	114636.00
18	35	8	0.7	270	250	122765.45	126415.67

Table 7.

Code	Variable	Low	High
A	Column Temp (°C)	30	55
B	Flow Rate (ml/min)	0.25	0.3
C	%B at 3min	30	65
D	%B at 4.1min	30	65
E	%B at 6min	30	65

Table 8.

Run	Column Temp (°C)	Flow Rate (ml/min)	%B at 3min	%B at 4.1min	%B at 6min
1	42.5	0.275	89.12	47.5	47.5
2	55	0.3	65	65	30
3	55	0.3	65	30	65
4	55	0.3	65	65	65
5	55	0.3	65	30	30
6	30	0.3	65	30	30
7	30	0.3	65	65	65
8	30	0.3	65	65	30
9	30	0.3	65	30	65
10	55	0.25	65	65	65
11	55	0.25	65	30	30
12	55	0.25	65	65	30
13	55	0.25	65	30	65
14	30	0.25	65	65	65
15	30	0.25	65	65	30
16	30	0.25	65	30	30
17	30	0.25	65	30	65
18	42.5	0.33	47.5	47.5	47.5
**19**	**72.23**	**0.275**	**47.5**	**47.5**	**47.5**
20	42.5	0.275	47.5	47.5	47.5
21	42.5	0.275	47.5	47.5	47.5
22	42.5	0.275	47.5	47.5	47.5
23	42.5	0.275	47.5	47.5	5.87
24	42.5	0.275	47.5	47.5	47.5
25	42.5	0.275	47.5	5.87	47.5

Run	Column Temp (°C)	Flow Rate (ml/min)	%B at 3min	%B at 4.1min	%B at 6min
26	42.5	0.275	47.5	89.12	47.5
27	42.5	0.275	47.5	47.5	47.5
28	42.5	0.275	47.5	47.5	47.5
29	42.5	0.275	47.5	47.5	47.5
30	42.5	0.275	47.5	47.5	89.12
31	42.5	0.275	47.5	47.5	47.5
32	12.76	0.275	47.5	47.5	47.5
33	42.5	0.21	47.5	47.5	47.5
34	55	0.3	30	65	30
35	55	0.3	30	30	65
36	55	0.3	30	65	65
37	55	0.3	30	30	30
38	30	0.3	30	65	30
39	30	0.3	30	30	30
40	30	0.3	30	65	65
41	30	0.3	30	30	65
42	55	0.25	30	65	30
43	55	0.25	30	65	65
44	55	0.25	30	30	30
45	55	0.25	30	30	65
46	30	0.25	30	30	30
47	30	0.25	30	30	65
48	30	0.25	30	65	30
49	30	0.25	30	65	65
50	42.5	0.275	5.87	47.5	47.5

Table 9.

Run	Response-1	Response-2	Response-3	Average	% RSD
1	82932	86111	86744	85262.33	2.40
2	78973	84240	80699	81304.00	3.30
3	81303	85590	87199	84697.33	3.60
4	86208	81061	84074	83781.00	3.09
5	90216	88064	97067	91782.33	5.12
6	74021	72828	74105	73651.33	0.97
7	72556	67535	68734	69608.33	3.77
8	74367	69842	69560	71256.33	3.79
9	73469	70939	70373	71593.67	2.30
10	87045	96733	93993	92590.33	5.39
11	102574	105280	106035	104629.67	1.74
12	105642	105491	104898	105343.67	0.37
13	110338	103202	102923	105487.67	3.98
14	84716	79978	78809	81167.67	3.85
15	80266	79475	82214	80651.67	1.75
16	89543	88485	86216	88081.33	1.93
17	82185	83127	79644	81652.00	2.21
18	82062	81976	79972	81336.67	1.45
**19**	**124903**	**127382**	**135592**	**129292.33**	**4.33**
20	93022	92334	88091	91149.00	2.93
21	90661	90635	91268	90854.67	0.39
22	89903	90693	91724	90773.33	1.01
23	94554	91908	105806	97422.67	7.57
24	94331	96247	91018	93865.33	2.82
25	97811	97305	99250	98122.00	1.03

Run	Response-1	Response-2	Response-3	Average	% RSD
26	91419	87881	88821	89373.67	2.05
27	87172	90085	94071	90442.67	3.83
28	94613	91629	93715	93319.00	1.64
29	95573	99410	95531	96838.00	2.30
30	84751	88884	88155	87263.33	2.53
31	88154	84730	88786	87223.33	2.50
32	79498	80641	79640	79926.33	0.78
33	108418	109105	109454	108992.33	0.48
34	107622	107172	105943	106912.33	0.81
35	111176	101900	105753	106276.33	4.38
36	99759	105890	99765	101804.67	3.48
37	114982	112762	110500	112748.00	1.99
38	87651	89161	88777	88529.67	0.89
39	89301	91764	91235	90766.67	1.43
40	79587	79064	78825	79158.67	0.49
41	81832	85386	86509	84575.67	2.89
42	122309	129132	131065	127502.00	3.61
43	119134	110448	112285	113955.67	4.02
44	122209	123820	126598	124209.00	1.79
45	123251	121678	127939	124289.33	2.62
46	102855	102108	100002	101655.00	1.46
47	94616	90741	92578	92645.00	2.09
48	98164	99762	94304	97410.00	2.88
49	93638	92723	86939	91100.00	3.99
50	106568	102875	105888	105110.33	1.87

Table 10.

A)

Source	Sequential p-value	Lack of Fit p-value	Adjusted R-Squared	Predicted R-Squared	
Linear	3.04826E-22	0.087736713	0.903670243	0.88553389	
2FI	0.366964338	0.08916514	0.90651774	0.877699184	
Quadratic	0.011241656	0.193371112	0.93263729	0.852448935	Suggested
Cubic	0.301197544	0.210339295	0.942375665	0.425126156	Aliased

B)

Source	Sum of Squares	df	Mean Square	F Value	p-value Prob > F	
Mean vs Total	4.43189E+11	1	4.43189E+11			
Linear vs Mean	9153523641	5	1830704728	92.93388053	3.04826E-22	
2FI vs Linear	216788234.5	10	21678823.45	1.134025286	0.366964338	
Quadratic vs 2FI	250482097.1	5	50096419.43	3.63666561	0.011241656	Suggested
Cubic vs Quadratic	234510925.6	15	15634061.71	1.326728714	0.301197544	Aliased
Residual	164974844.9	14	11783917.49			
Total	4.53209E+11	50	9064189989			

C)

Source	Sum of Squares	df	Mean Square	F Value	p-value (Prob > F)	
Linear	809642496.8	37	21882229.64	2.681946037	0.087736713	
2FI	592854262.3	27	21957565.27	2.691179378	0.08916514	
Quadratic	342372165.2	22	15562371.14	1.907366859	0.193371112	Suggested
Cubic	107861239.6	7	15408748.51	1.888538448	0.210339295	Aliased
Pure Error	57113605.33	7	8159086.476			

D)

Source	Std.Dev.	R-Squared	Adjusted R-Squared	Predicted R-Squared	PRESS	
Linear	4438.355813	0.91349981	0.903670243	0.88553389	1146982441	
2FI	4372.265087	0.935134758	0.90651774	0.877699184	1225488392	
Quadratic	3711.518745	0.960132274	0.93263729	0.852448935	1478502949	Suggested
Cubic	3432.77111	0.983535904	0.942375665	0.425126156	5760396737	Aliased

Table 11.

ANOVA for Response Surface Quadratic model						
**Analysis of variance table [Partial sum of squares - Type III]**						
**Source**	**Sum of Squares**	**df**	**Mean Square**	**F value**	**p-value Prob > F**	
Model	9.626E+009	21	4.584E+008	32.54	< 0.0001	Significant
A-Column Temp	4.495E+009	1	4.495E+009	319.07	< 0.0001	Significant
B-Flow Rate	1.806E+009	1	1.806E+009	128.22	< 0.0001	Significant
C-%B at 3min	2.416E+009	1	2.416E+009	171.48	< 0.0001	Significant
D-%B at 4.1min	1.766E+008	1	1.766E+008	12.54	0.0014	Significant
E-%B at 6min	2.605E+008	1	2.605E+008	18.49	0.0002	Significant
AB	5.908E+007	1	5.908E+007	4.19	0.0501	Not significant
AC	1.121E+008	1	1.121E+008	7.96	0.0087	Significant
AD	7.208E+006	1	7.208E+006	0.51	0.4803	Not significant
AE	34300.17	1	34300.17	2.435E-003	0.9610	Not significant
BC	3.085E+006	1	3.085E+006	0.22	0.6434	Not significant
BD	20418.84	1	20418.84	1.450E-003	0.9699	Not significant
BE	3.878E+006	1	3.878E+006	0.28	0.6039	Not significant
CD	8.066E+005	1	8.066E+005	0.057	0.8126	Not significant
CE	2.776E+007	1	2.776E+007	1.97	0.1714	Not significant
DE	2.783E+006	1	2.783E+006	0.20	0.6601	Not significant
A^2	2.389E+008	1	2.389E+008	16.96	0.0003	Significant
B^2	9.065E+006	1	9.065E+006	0.64	0.4292	Not significant
C^2	9.239E+006	1	9.239E+006	0.66	0.4249	Not significant
D^2	1.309E+006	1	1.309E+006	0.093	0.7628	Not significant
E^2	5.003E+005	1	5.003E+005	0.036	0.8519	Not significant
Residual	3.944E+008	28	1.409E+007			
Lack of Fit	3.373E+008	21	1.606E+007	1.97	0.1818	Not significant
Pure Error	5.711E+007	7	8.159E+006			

Figure. 8

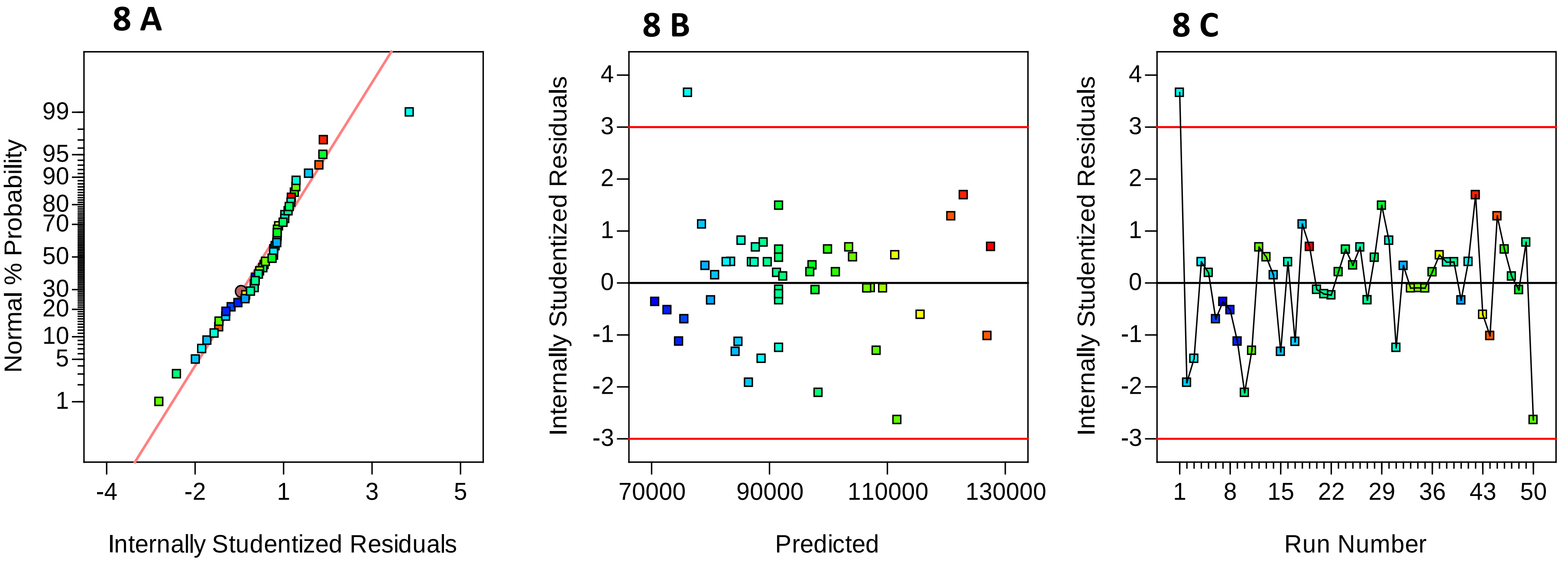

8 A
Normal % Probability
99
95
90
80
70
50
30
20
10
5
1
-4
-2
1
3
5
Internally Studentized Residuals
8 B
Internally Studentized Residuals
4
3
2
1
0
-1
-2
-3
70000
90000
110000
130000
Predicted
8 C
Internally Studentized Residuals
4
3
2
1
0
-1
-2
-3
1
8
15
22
29
36
43
50
Run Number

Figure. 9

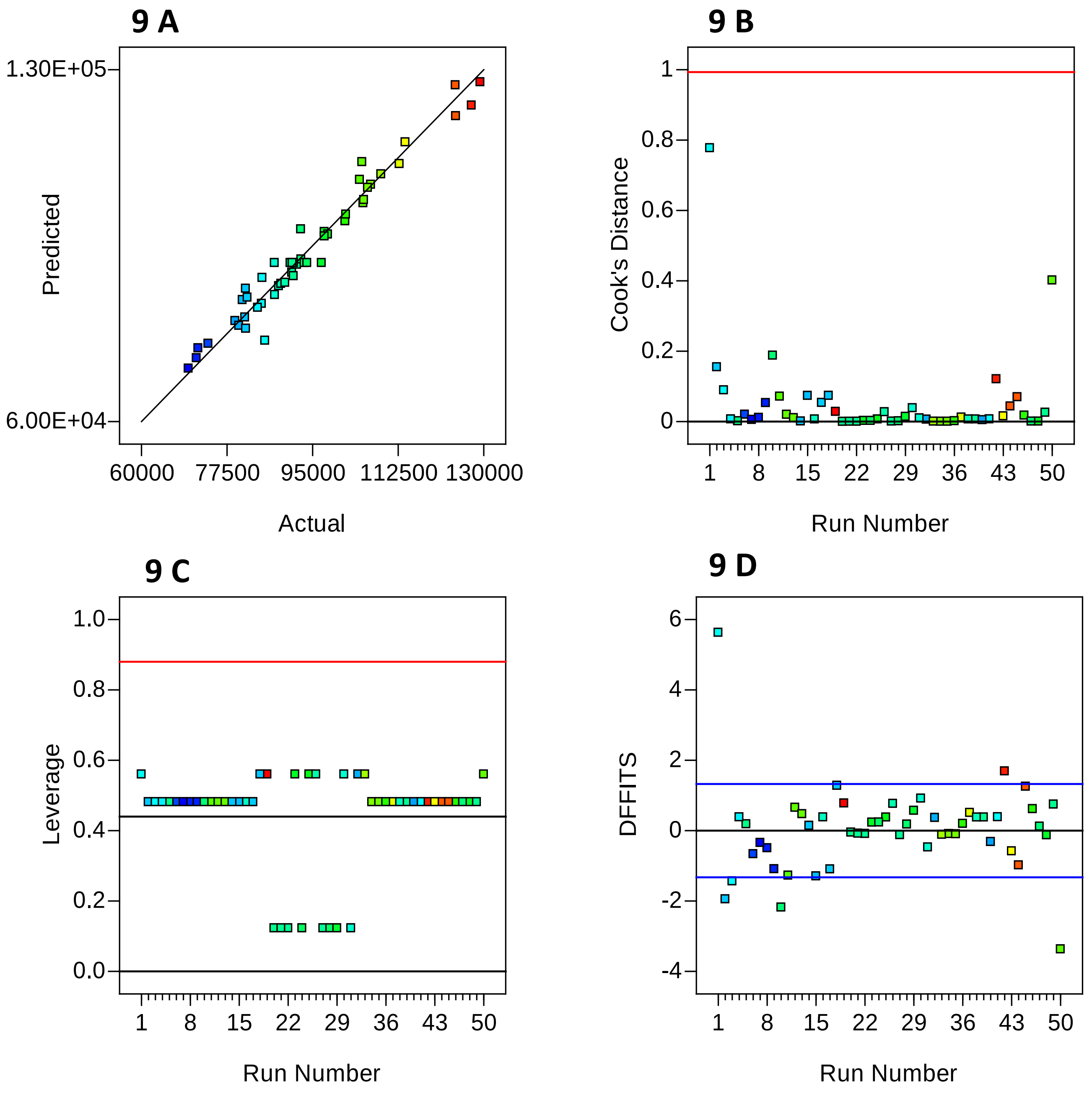

9 A
1.30E+05
6.00E+04
Predicted
60000
77500
95000
112500
130000
Actual
9 B
1
0.8
0.6
0.4
0.2
0
Cook's Distance
1
8
15
22
29
36
43
50
Run Number
9 C
1.0
0.8
0.6
0.4
0.2
0.0
Leverage
1
8
15
22
29
36
43
50
Run Number
9 D
6
4
2
0
-2
-4
DFFITS
1
8
15
22
29
36
43
50
Run Number

Figure. 10

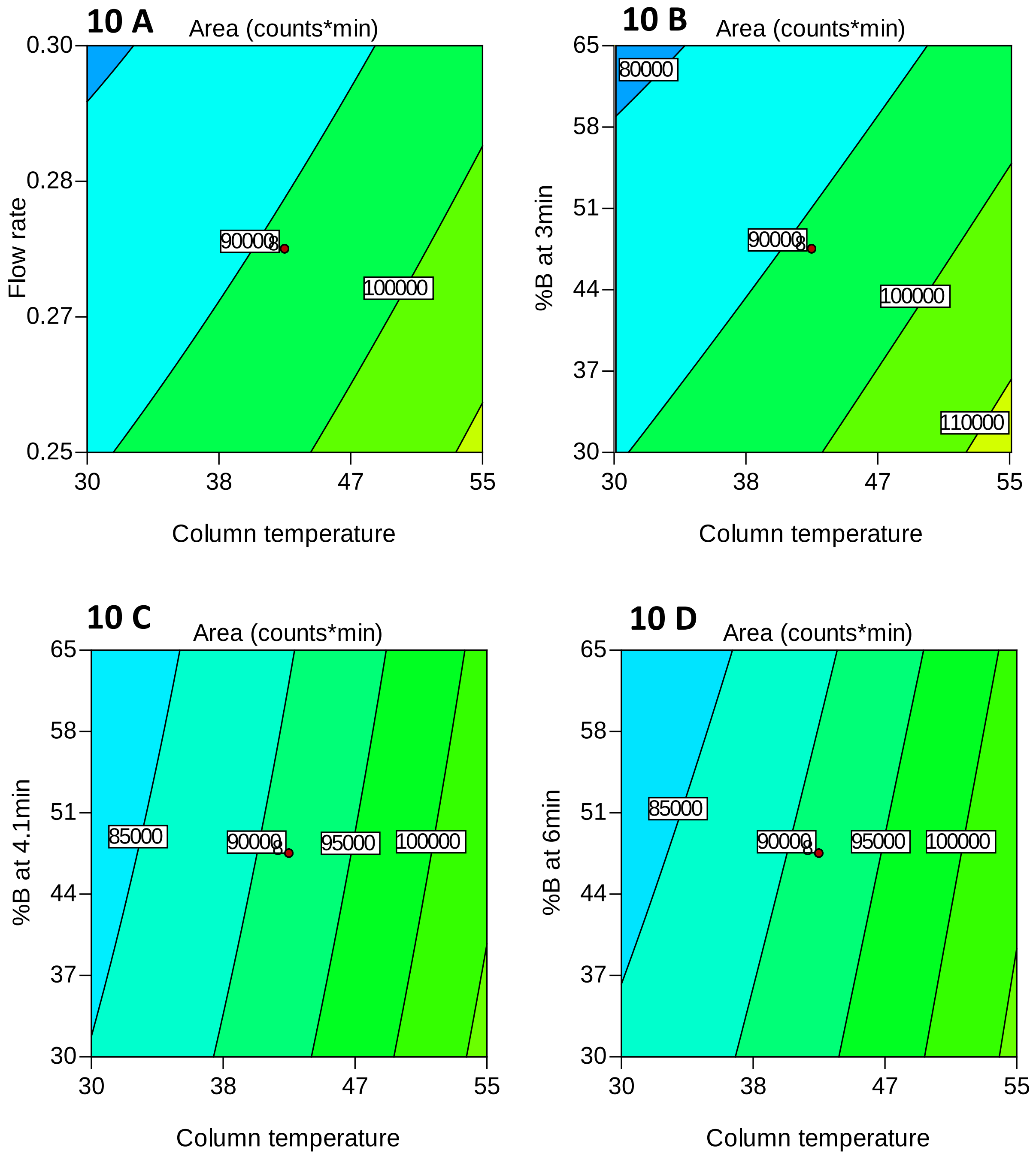
10 A
Area (counts*min)
Flow rate
0.30
0.28
0.27
0.25
90000
100000
8
30
38
47
55
Column temperature
10 B
Area (counts*min)
%B at 3min
65
58
51
44
37
30
80000
90000
100000
110000
8
30
38
47
55
Column temperature
10 C
Area (counts*min)
%B at 4.1min
65
58
51
44
37
30
85000
90000
95000
100000
8
30
38
47
55
Column temperature
10 D
Area (counts*min)
%B at 6min
65
58
51
44
37
30
85000
90000
95000
100000
8
30
38
47
55
Column temperature

Figure. 11

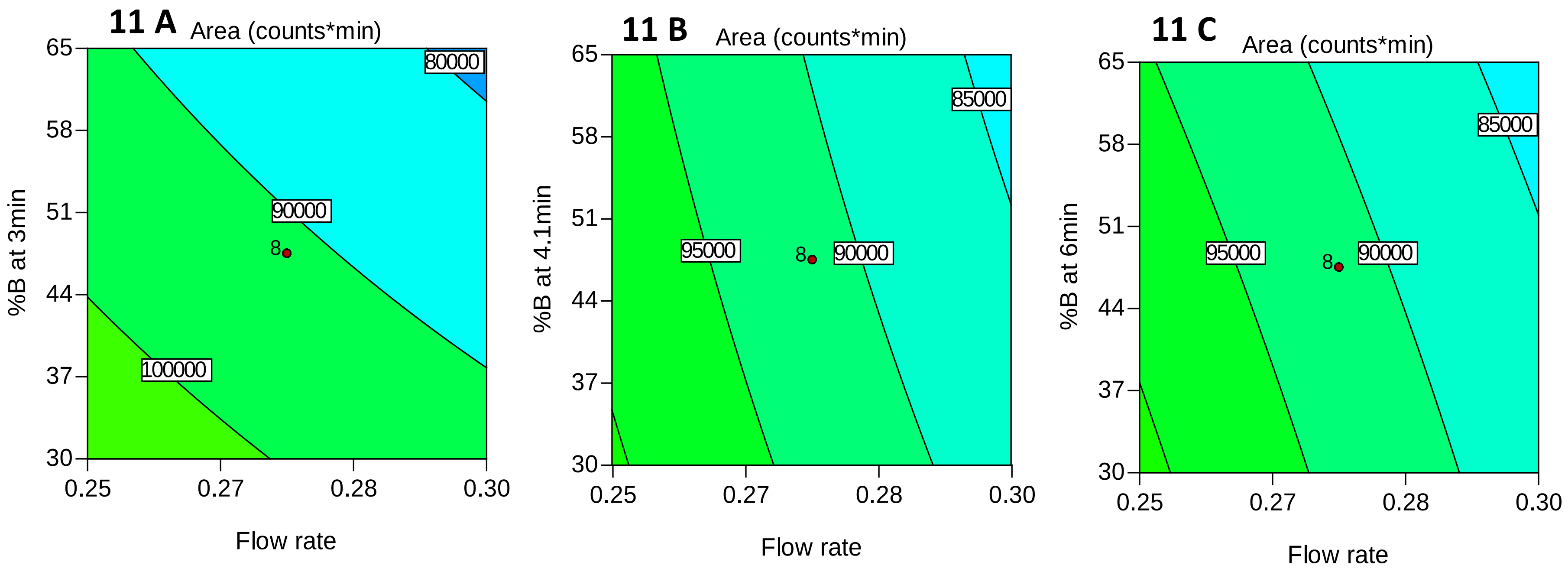

11 A
Area (counts*min)
65
58
51
44
37
30
80000
90000
8
100000
0.25
0.27
0.28
0.30
%B at 3min
Flow rate
11 B
Area (counts*min)
65
58
51
44
37
30
85000
95000
8
90000
0.25
0.27
0.28
0.30
%B at 4.1min
Flow rate
11 C
Area (counts*min)
65
58
51
44
37
30
85000
95000
8
90000
0.25
0.27
0.28
0.30
%B at 6min
Flow rate

Figure. 12

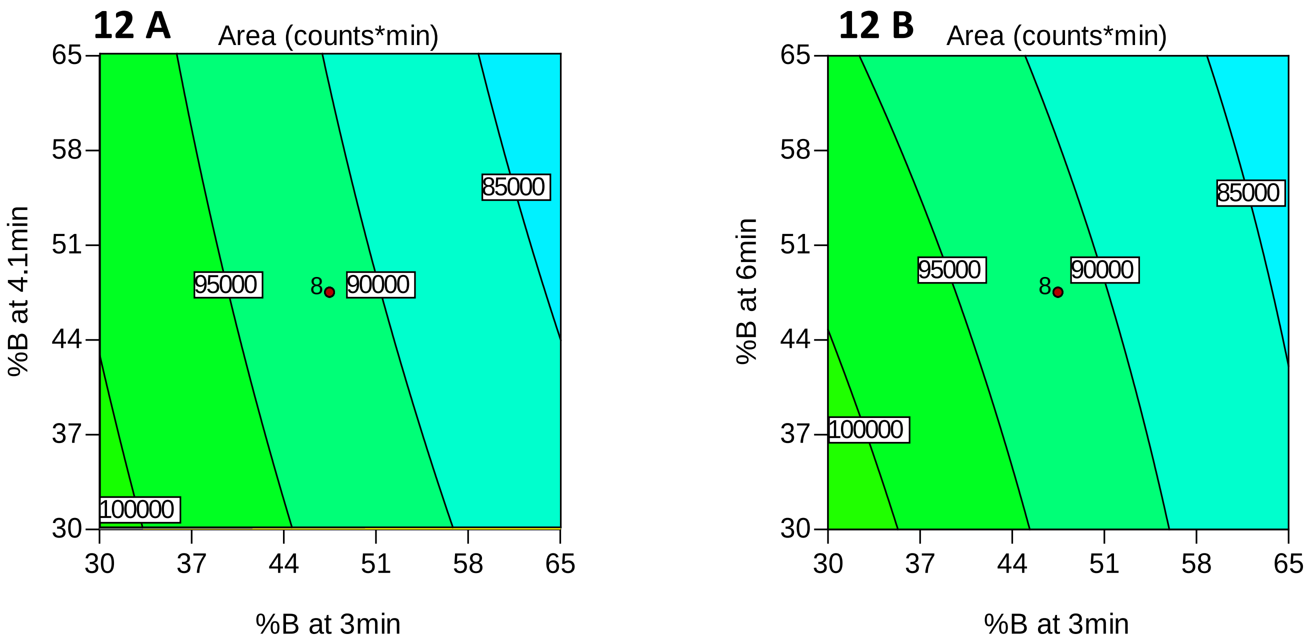

12 A
Area (counts*min)
%B at 4.1min
%B at 3min
65
58
51
44
37
30
30
37
44
51
58
65
85000
95000
90000
8
100000
12 B
Area (counts*min)
%B at 6min
%B at 3min
65
58
51
44
37
30
30
37
44
51
58
65
85000
95000
90000
8
100000

Figure. 13

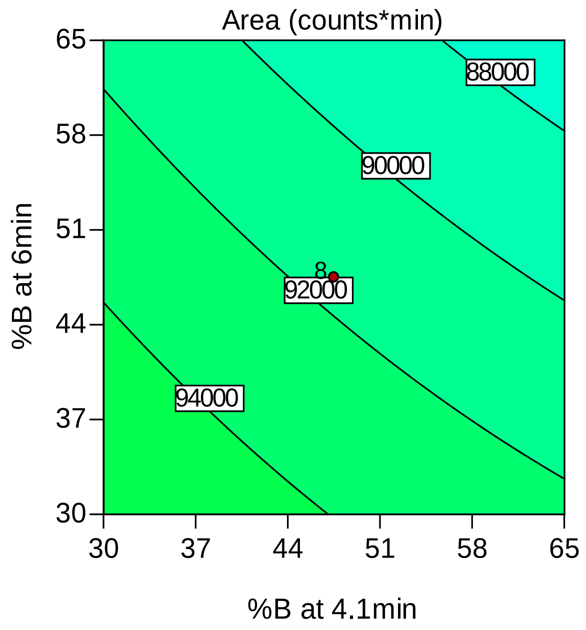
Area (counts*min)
65
58
51
44
37
30
%B at 6min
88000
90000
92000
94000
8
30
37
44
51
58
65
%B at 4.1min

Table. 12

Prediction Run	Column Temp (^{0}C)	Flow Rate (mL/min)	%B at 3min	%B at 4.1min	%B at 6min	Predicted Area	Experimental Area (EA)	EA %RSD
**1**	**70**	**0.22**	**6**	**85**	**6**	**187464.01**	**143542.00**	**2.34**
2	70	0.25	10	85	10	172043.19	134888.00	2.84
3	70	0.3	10	85	20	150306.58	124599.33	1.52
**4**	**72**	**0.275**	**47.5**	**47.5**	**47.5**	**156284.32**	**135711.00**	**2.01**
5	60	0.275	20	85	15	140603.19	119764.33	1.34
6	50	0.22	10	85	10	148162.10	137895.66	5.72
7	40	0.25	35	85	35	112932.47	120031.33	1.33
8	30	0.25	15	85	15	111274.78	113412.00	3.43
9	30	0.25	35	85	35	102438.18	112510.00	0.92
10	30	0.25	6	85	6	116311.67	116579.00	1.79

Figure. 14

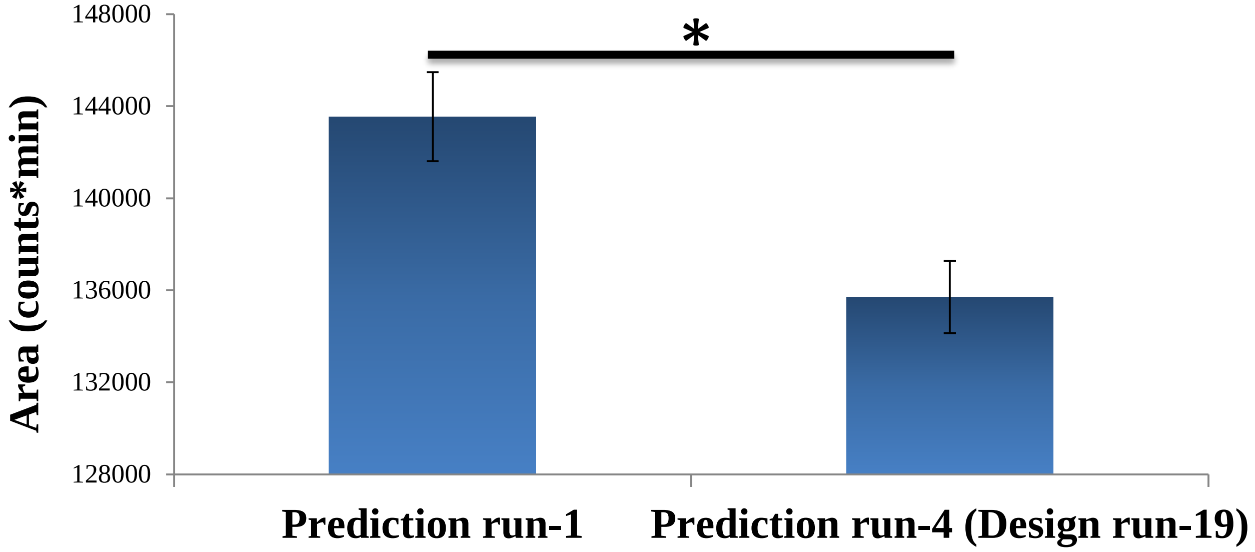

**Unpaired t test**	
P value	0.0350
P value summary	*
Are means signif. different? (P < 0.05)	Yes
One- or two-tailed P value?	Two-tailed
t, df	t=3.136 df=4

Table. 13

Method	VT	ITT	SGP	AGP	SWGP
Gen-MS-I	250	200	25	2	0.5
CCD-MS	270	301	36	7.5	1
CCD-LC	270	301	36	7.5	1

Table. 14

Method	Column Temperature	Flow rate (ml/min)	Time (min)	%B (Acetonitrile)
Gen-MS-I	30	0.25	0 to 4	50 to 80
		0.25	4 to 8	80 to 50
		0.25	8 to 15	50 to 50
CCD-MS	30	0.25	0 to 4	50 to 80
		0.25	4 to 8	80 to 50
		0.25	8 to 15	50 to 50
CCD-LC	70	0.25	0 to 3	50 to 6
		0.22	3 to 4.1	6 to 85
		0.22	4.1 to 6	85 to 6
		0.25	6 to 8	6 to 50
